# MOF‐Derived Cu‐TiO_2_/Alginate Micromotors for Buoyancy‐Regulated 3D Motion

**DOI:** 10.1002/advs.77408

**Published:** 2026-08-27

**Authors:** Wei Zou, Qing Wu, Jiawei Lin, Kunchen Li, Yulong Ying, Josep Puigmartí‐Luis, Salvador Pané, Sheng Wang

**Affiliations:** ^1^ School of Materials Science and Engineering Zhejiang Sci‐Tech University Hangzhou People's Republic of China; ^2^ Departament De Ciència dels Materials i Química Física Institut De Química Teòrica i Computacional University of Barcelona (UB) Barcelona Spain; ^3^ Institució Catalana de Recerca i Estudis Avançats (ICREA) Barcelona Spain; ^4^ Multi‐Scale Robotics Lab Institute of Robotics and Intelligent Systems ETH Zürich Zürich Switzerland

**Keywords:** 3D motion, buoyancy regulation, micromotors, microvesicles, MOFs

## Abstract

Micro‐ and nanomotors (MNMs) promise impact in the areas of environmental remediation and biomedicine, but reliable three‐dimensional (3D) control remains challenging. Conventional bubble‐propelled MNMs can move in 3D, yet their trajectories are stochastic and energy‐inefficient. Here we introduce a buoyancy‐regulated strategy inspired by cyanobacteria‐like microvesicles. We engineer sodium‐alginate (SA)hydrogel magnetic micromotors (SA/Cu‐TiO_2_/Fe_3_O_4_), in which Cu‐TiO_2_ is derived from the Ti‐based metal–organic framework MIL‐125(Ti_v_), yielding a porous and defect‐rich photocatalyst with uniformly distributed Cu sites for enhanced H_2_O_2_ decomposition and photocatalytic activity compared with pristine TiO_2_. Upon illumination, Cu‐TiO_2_ catalyzes H_2_O_2_ decomposition to generate O_2_ pockets that are retained within the elastic, semipermeable alginate matrix, enabling programmable flotation and precise control of vertical (*Z*‐axis) position as part of full 3D navigation. We analyze the energetics and cross‐scale mass‐transport mechanisms governing this buoyancy control, and we demonstrate guided 3D motion through a maze via magnetic steering. In model turbid water, the micromotors achieve high removal performance across diverse organic contaminants. This bioinspired platform provides a prototype and motion‐control paradigm for 3D‐motile MNMs, advancing practical remediation in aquatic environments.

## Graphical abstract

In this work, cyanobacteria‐inspired sodium alginate (SA)/Cu‐TiO_2_/Fe_3_O_4_ hydrogel micromotors generate and encapsulate O_2_ microvesicles upon light irradiation, enabling buoyancy‐mediated *Z*‐axis upward migration and surface enrichment at the light‐exposed water‐air surface. The synergistic combination of intensified light harvesting, efficient transmembrane pollutant diffusion, and concurrent photocatalytic/Fenton‐like reactive oxygen species (ROS) generation endows the micromotors with outstanding degradation capacity toward various organic pollutants, paving a feasible pathway for practical remediation of turbid, complicated wastewater systems.

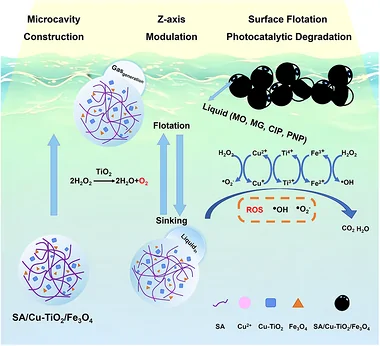

## 1 Introduction

Motion is a fundamental feature of life for organisms and collectives. Behaviors include obstacle avoidance [1], hunting [2], foraging, seeking shelter [3, 4], and fertilization [5]. Across various length scales, organisms inhabiting liquid environments have evolved diverse strategies to optimize locomotion according to their specific physiological characteristics. For instance, micro‐organisms commonly employ motile organelles such as cilia, flagella, and gas vesicles for propulsion [6, 7], whereas larger aquatic organisms, such as fish, rely on fins for maneuverability and swim bladders for buoyancy regulation [8]. Based on these observations, we propose that motility in liquid environments can be broadly categorized into two principal mechanisms, i.e., flagellar or ciliary movement, and buoyancy regulation in gas containers.

Micro‐ and nanomotors (MNMs) are artificial micro‐ and nanoscale devices that have the ability to navigate in fluids by means of external energy inputs, such as chemical fuels, electric and magnetic fields, light, and ultrasound [9]. While many MNMs have demonstrated effective motion in two‐dimensional or near‐planar environments, realizing controllable three‐dimensional (3D) motion remains significantly more challenging. This is because 3D locomotion requires not only forward propulsion, but also precise regulation of vertical displacement, orientation, and trajectory in complex fluid environments. To date, the primary strategies for enabling 3D motion include bubble propulsion [10, 11, 12], light [13, 14, 15], magnetic field‐ [16] and electric field‐based control [17]. Among externally actuated systems, magnetically driven MNMs are particularly attractive for biomedical applications because they offer rapid response, wireless control, deep tissue penetration, and generally good biocompatibility, making them highly promising for controlled 3D motion. However, in open‐space environments, sustained 3D locomotion still generally relies on continuous external actuation and relatively complex field‐control setups to maintain vertical positioning and trajectory. In light of these limitations, increasing attention has been directed toward complementary bioinspired strategies, such as gas‐vesicle‐inspired buoyancy regulation, as exemplified by highly efficient natural systems like cyanobacteria.

Cyanobacteria are specialized algae containing intracellular microvesicles and phycocyanin [18, 19]. These microvesicles enable cyanobacteria to regulate their buoyancy by modulating microvesicle volume, allowing them to reposition vertically in aquatic environments to optimize access to light and nutrients distributed along the water column [20, 21]. Compared to flagellar propulsion, which is commonly used for suspension and vertical movement in water, microvesicle‐mediated buoyancy regulation demonstrates significantly higher energy efficiency. Fluid dynamic analyses reveal that the energy conversion efficiency of flagellar‐driven microbial motion is as low as 1–2%, with the remaining 98% dissipated as heat [22]. In contrast, buoyancy regulation via microvesicles represents a fundamentally distinct mechanism from traditional bubble‐propelled MNMs. In bubble‐recoil systems, gas bubbles form on the surface of the micromotor and generate thrust through random detachment. By comparison, buoyancy‐regulated systems confine bubbles within the microvesicle structure, adjusting internal gas volume in response to environmental stimuli to achieve controlled *Z*‐axis displacement. Although buoyancy‐based locomotion has been demonstrated in some macroscopic and microscale systems, the bubbles in these designs are typically generated at the fluid‐surface interface of the structures, and exhibit poor stability and uncontrolled detachment [23, 24, 25]. To date, no synthetic MNM has successfully incorporated an internalized, vesicle‐like component that mimics the behavior of natural cyanobacterial microvesicles. In our investigation, we identified sodium alginate hydrogel (SA) as an ideal candidate material for constructing such microvesicles‐mimetic structures in MNMs, enabling biologically inspired buoyancy modulation.

Here, we developed SA/Cu‐TiO_2_/Fe_3_O_4_ micromotors with cyanobacteria‐inspired functionalities by integrating SA, a metal–organic framework (MOF)‐derived photocatalyst (Cu‐TiO_2_), and magnetic particles (Fe_3_O_4_). Specifically, Cu‐TiO_2_ was obtained by calcination of Cu‐modified MIL‐125(Ti_v_), a Ti‐based metal–organic framework used as a precursor to construct porous and defect‐rich Cu‐doped TiO_2_ with uniformly dispersed active sites. Compared with other TiO_2_, this MOF‐derived catalyst offers improved charge separation, enhanced H_2_O_2_ decomposition, and higher photocatalytic activity, making it particularly suitable for efficient in situ O_2_ generation. By precisely modulating the light intensity and exposure duration in H_2_O_2_ solution, we achieve dynamic construction and adjustment of a microvesicle‐like structure, enabling controlled *Z*‐axis propulsion. The interfacial mass transport mechanism at the microvesicle boundary was explored through in situ fluorescence experiments, providing a comprehensive explanation of the *Z*‐axis motion behavior. Under magnetic guidance, the micromotors demonstrated agile 3D maneuverability in a 3D maze. Notably, in 0.03 wt.% H_2_O_2_, the SA/Cu‐TiO_2_/Fe_3_O_4_ micromotors rapidly generated microvesicles and exhibited upward buoyant motion with excellent floating efficiency of 72.4%, even after 24 h in dark conditions. In turbid water, the micromotors ascended from the bottom to the surface within 120 s, facilitating enhanced photocatalytic performance. In practical degradation experiments, the floating micromotors achieved 90.3% removal efficiency for methyl orange (MO) within 240 min, which was 1.96 times higher than in the settling state. Our finding provides both theoretical insight and practical guidance for the design of novel 3D‐motile MNMs inspired by cyanobacteria microvesicle dynamics, contributing to the development of effective strategies for aquatic environmental remediation.

## 2 Results and Discussion

### 2.1 Synthesis and Characterization of SA/Cu‐TiO_2_/Fe_3_O_4_ Micromotors

**FIGURE 1:**
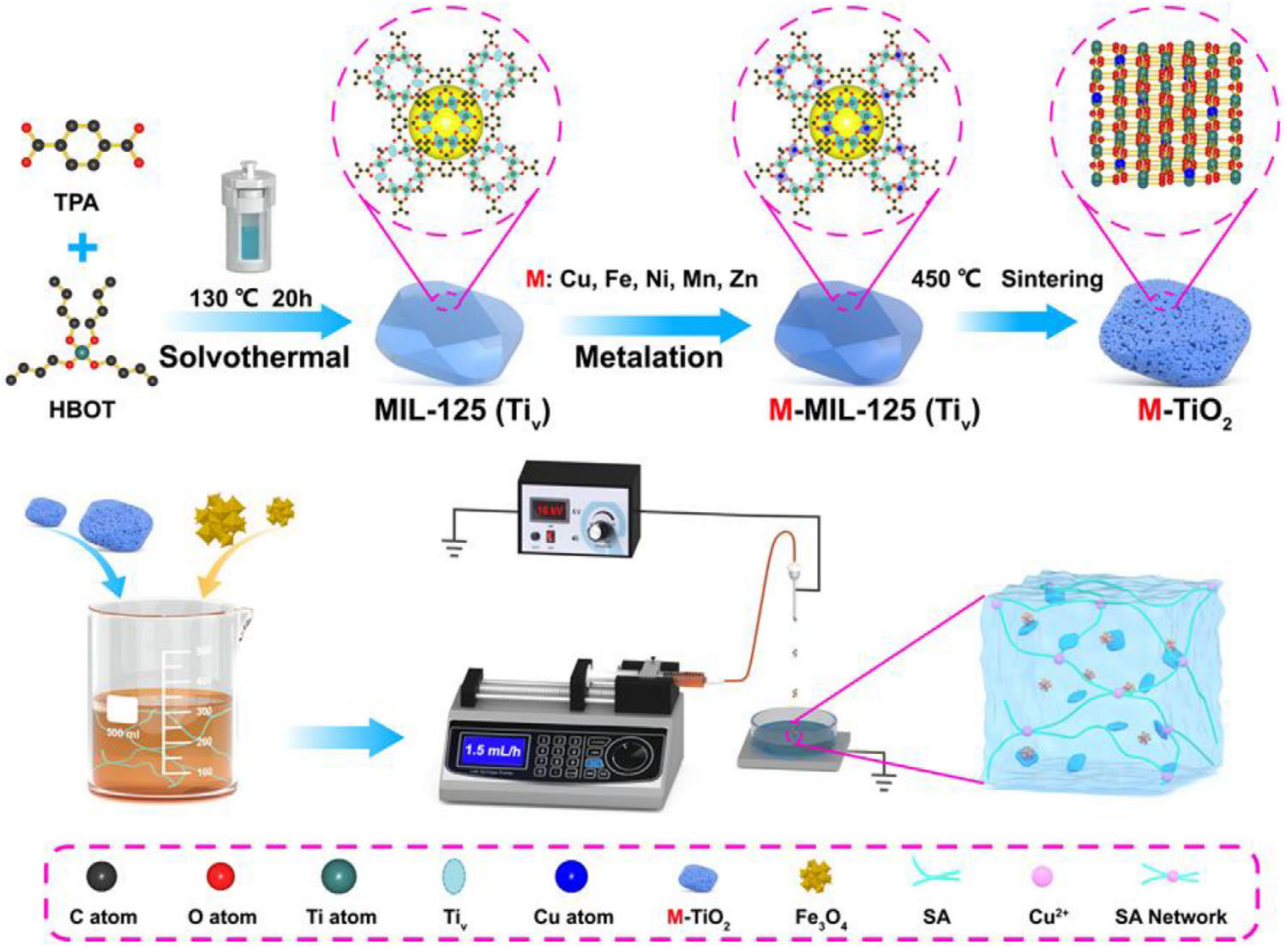
Schematic illustration of the synthesis process for metallized TiO_2_ and the fabrication of SA/Cu‐TiO_2_/Fe_3_O_4_ micromotors using electrostatic spraying.

MIL‐125(Ti_v_) was prepared using a modified solvothermal method based on previously reported literature [26]. Briefly, terephthalic acid (PTA) was dissolved in N, N‐dimethylformamide (DMF), followed by the addition of methanol and titanium butoxide (C_16_H_36_O_4_Ti) to form a homogeneous solution. The mixture was then subjected to solvothermal reaction in an autoclave reactor, yielding white crystalline MIL‐125(Ti_v_). The resulting MIL‐125(Ti_v_) was dispersed in aqueous solutions of various metal ions (Cu^2+^, Fe^3+^, Ni^2+^, Mn^2+^, and Zn^2+^) and stirred at room temperature for 3 h to obtain metal‐doped MIL‐125(Ti_v_) (M‐MIL‐125(Ti_v_), M═Cu, Fe, Ni, Mn, and Zn). Both MIL‐125(Ti_v_) and M‐MIL‐125(Ti_v_) were subsequently annealed at 450°C for 4 h in a muffle furnace to produce TiO_2_ and M‐TiO_2_, respectively. The Cu‐TiO_2_ used in this study is the one containing 1.52 at% Cu. To fabricate the micromotors, Cu‐TiO_2_ and Fe_3_O_4_ nanoparticles (NPs) were dispersed in sodium alginate (SA) solution. The hydrogel‐based SA/Cu‐TiO_2_/Fe_3_O_4_ micromotors were then obtained using the electrospraying technique (Figure 1) [27].

**FIGURE 2:**
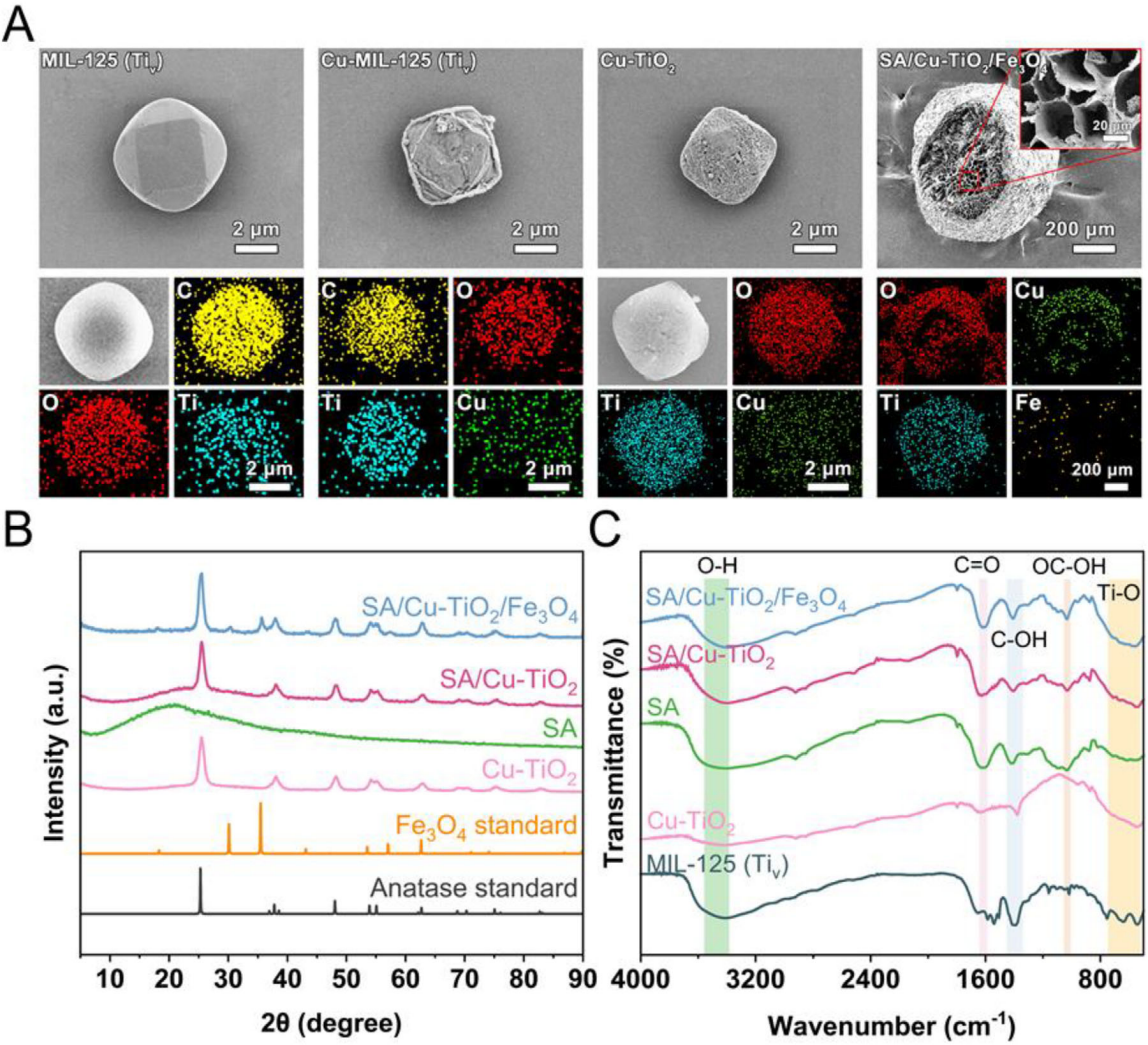
Morphological and structural characterization of SA/Cu‐TiO_2_/Fe_3_O_4_ micromotors. (A) SEM images of MIL‐125(Ti_v_), Cu‐MIL‐125(Ti_v_), Cu‐TiO_2_, and SA/Cu‐TiO_2_/Fe_3_O_4_ micromotors, along with corresponding elemental distribution mapping images showing the spatial distribution of C, O, Ti, Cu, and Fe elements. (B) XRD patterns of SA/Cu‐TiO_2_/Fe_3_O_4_ micromotors. (C) FTIR spectra of SA/Cu‐TiO_2_/Fe_3_O_4_ micromotors.

The scanning electron microscopy (SEM) and energy‐dispersive X‐ray spectroscopy (EDS) images of MIL‐125(Ti_v_), Cu‐MIL‐125(Ti_v_), Cu‐TiO_2_, and SA/Cu‐TiO_2_/Fe_3_O_4_ micromotors are shown in Figure 2A. The pristine MIL‐125(Ti_v_) exhibited a regular, smooth, cake‐like morphology with a uniform size of approximately 4.5 µm. Cu‐MIL‐125(Ti_v_) and Cu‐TiO_2_ retained the overall morphology of MIL‐125(Ti_v_), but displayed more obvious metallic features on the surface. Notably, the surface of annealed Cu‐TiO_2_ became rougher, as high‐temperature annealing altered the original carbon framework and reduced the densification of Cu‐TiO_2_ [28]. The EDS in Figure 2A confirms the successful doping of the metallic element Cu into M‐MIL‐125 (Ti_v_). Figures S1 and S2 further demonstrate that different contents of Cu, as well as other metal elements (Fe, Ni, Mn, and Zn), were successfully doped into M‐MIL‐125 (Ti_v_) and M‐TiO_2_, respectively. The SA/Cu‐TiO_2_ and SA/Cu‐TiO_2_/Fe_3_O_4_ micromotors preserved the porous hydrogel structure (Figure S3), facilitating efficient water and gas storage. Unless otherwise specified, M‐TiO_2_ containing 1.52 at% Cu is referred to as Cu‐TiO_2,_ while samples with other copper contents are designated as Cu_n_‐TiO_2_. The microstructures of commercial P25, structurally stable spherical TiO_2_ (TiO_2_‐s), and Cu‐TiO_2_ were characterized via transmission electron microscopy (TEM). TEM results reveal that P25 mainly consists of irregular nanoparticles, TiO_2_‐s presents a well‐defined spherical morphology, and Cu species are atomically dispersed as single atoms in Cu‐TiO_2_ (Figures S4–S6). The corresponding x‐ray diffraction (XRD) patterns of the SA/Cu‐TiO_2_/Fe_3_O_4_ and M‐TiO_2_ micromotors are shown in Figure 2B and Figure S7. Characteristic diffraction peaks of Fe_3_O_4_ and anatase were detected in the SA/Cu‐TiO_2_/Fe_3_O_4_ micromotors, indicating the successful introduction of Fe_3_O_4_ and anatase into the micromotors, whereas pure SA did not exhibit any obvious diffraction peaks, which is in agreement with that reported in the literature [29]. X‐ray photoelectron spectroscopy (XPS) analysis revealed the chemical state and coordination environment of Cu atoms (Figure S8). Subsequently, Fourier‐transform infrared (FTIR) spectroscopy was performed to further analyze the samples (Figure 2C). The FTIR spectra confirmed that sodium alginate (SA) served as the primary polymer backbone in the SA/Cu‐TiO_2_/Fe_3_O_4_ micromotors. A broad absorption band at 3445 cm^−1^ was assigned to the O─H stretching vibration, corresponding to SA. The peak at 1625 cm^−1^ corresponds to the asymmetric stretching vibration of C═O in SA, while the peak at 1430 cm^−1^ was attributed to the C─OH stretching, and the peak at 1024 cm^−1^ was assigned to the symmetric C─O─C stretching [30]. These distinctive absorption peaks were observed in SA and SA/Cu‐TiO_2_/Fe_3_O_4_ micromotors. The SA/Cu‐TiO_2_/Fe_3_O_4_ micromotors were observed to show a characteristic peak of Ti─O at 651 cm^−1^ [31] which was not observed with pure SA, thus confirming the successful incorporation of Cu‐TiO_2_ into the SA matrix.

### 2.2 Motion of M‐TiO_2_ Under Light

TiO_2_ is a widely used photocatalyst and plays a central role in the design of light‐driven MNMs. However, commercial TiO_2_ (e.g., P25) exhibits strong absorption only in the ultraviolet region, significantly limiting its practical applications. In contrast, TiO_2_ prepared from MIL‐125(Ti_v_) displays enhanced light absorption properties, particularly when doped with metal atoms (M‐TiO_2_), which introduce strong absorption features in the visible light range (the instantaneous photocurrent responses of M‐TiO_2_ are presented in Figures S9–S11 and Movies S1–S3). To explore the propulsion performance of these materials, we systematically evaluated the kinematic behavior of M‐TiO_2_ under different H_2_O_2_ aqueous solution conditions, using H_2_O_2_ as chemical fuel. Upon light irradiation, Cu‐TiO_2_ in H_2_O_2_ undergoes the following reactions (Equations (1), (2), (3), (4), (5)):

(1) $$\mathrm{Cu}-\mathrm{Ti}O_{2}+\mathrm{hv}\to h^{+}+e^{-}$$

(2) $$Cu^{2+}+e^{-}\to Cu^{+}$$

(3) $$Cu^{+}+h^{+}\to Cu^{2+}$$

(4) $$H_{2}O_{2}+2h^{+}\to O_{2}+2H^{+}$$

(5) $$H_{2}O_{2}+2e^{-}+2H^{+}\to 2H_{2}O$$

**FIGURE 3:**
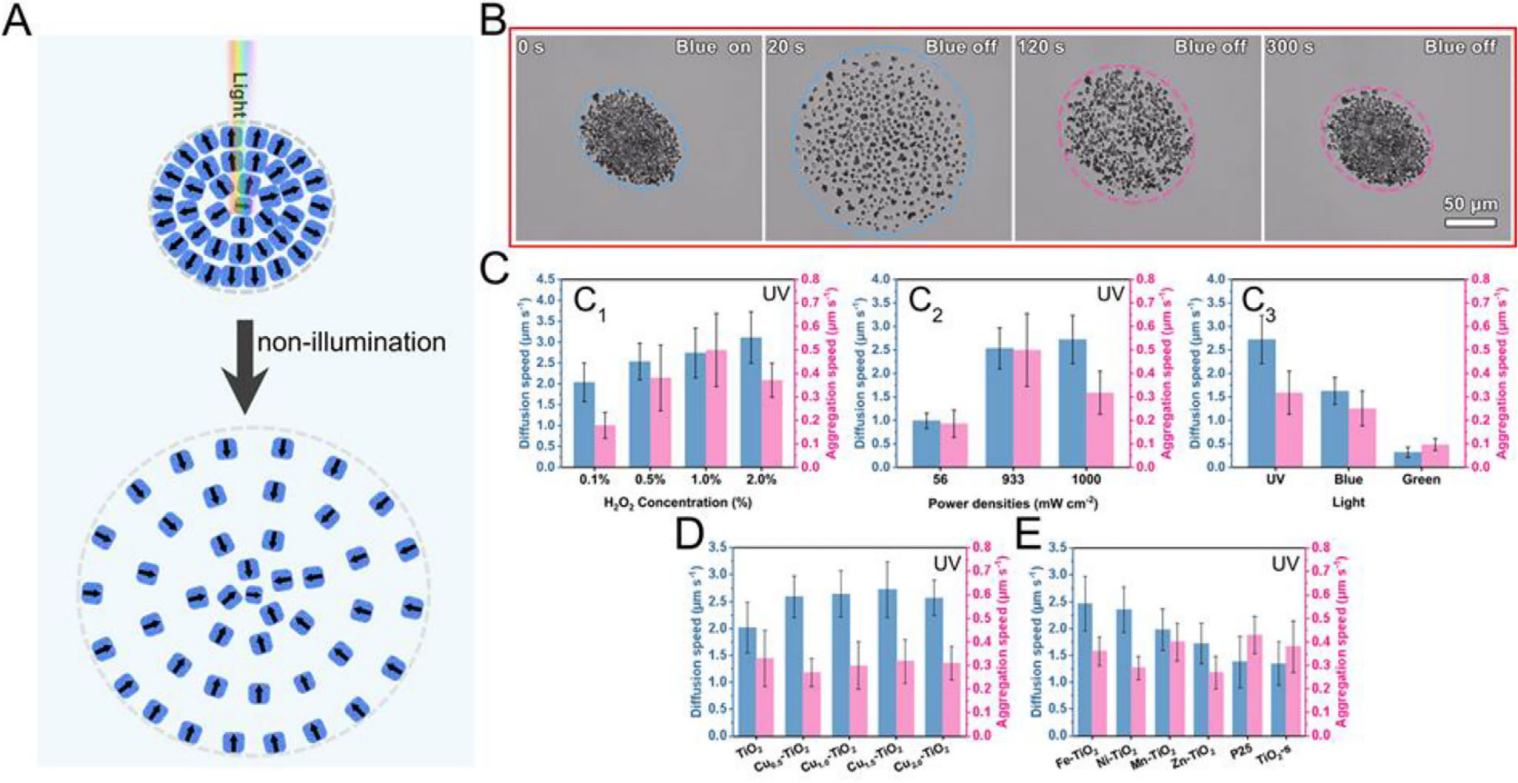
Light‐responsive behavior and photocatalytic performance of Cu‐TiO_2_ NPs. (A) Schematic illustration of the mechanism underlying negative phototaxis of a Cu‐TiO_2_ swarm under a light gradient. (B) Time‐sequenced images showing the light‐induced diffusion and aggregation of Cu‐TiO_2_ in 1.0 wt.% H_2_O_2_ solution under blue‐light irradiation (220 mW cm^−2^) and in the dark (Movie S3). The dashed line indicates the swarm boundary. (C) Comparison of diffusion and aggregation speeds of Cu‐TiO_2_ under different conditions: (C_1_) different H_2_O_2_ concentrations under UV light (1000 mW cm^−2^), (C_2_ and C_3_) different power densities and wavelengths in 1.0 wt.% H_2_O_2_ solution. (D, E) Diffusion and aggregation speed of M‐TiO_2_, commercial P25, and TiO_2_‐s under UV‐light irradiation.

Cu‐TiO_2_ exhibits negative phototropism in H_2_O_2_ aqueous solution under light irradiation, with particles migrating away from the light source (Figure 3A) [32]. This behavior originates from the asymmetric ion distribution and local potential gradient around Cu‐TiO_2_ particles [33]. In the dark, the difference in H^+^/OH^−^ diffusion and the hindered diffusion of OH^−^ near Cu‐TiO_2_ promote particle aggregation. Upon light irradiation, photocatalytic reactions on the Cu‐TiO_2_ surface generate H^+^ and O_2_, establishing outward electrolyte and non‐electrolyte concentration gradients. Under the combined effect of these gradients, Cu‐TiO_2_ clusters diffuse away from the light source [34, 35, 36]. When the light is switched off, the concentration of O_2_ rapidly decreases, and no new H^+^ is produced. However, the slower‐diffusing OH^−^, still hindered by Cu‐TiO_2_, accumulates near the particle surface. This re‐establishes a local potential gradient between distant H^+^ ions and Cu‐TiO_2_ particles, promoting reaggregation of Cu‐TiO_2_ clusters.

Initially, Cu‐TiO_2_ particles were in an aggregated state. Upon light irradiation, they underwent rapid diffusion, expanding to about 2.1 times their original size within 20 s. When the light source was switched off, the Cu‐TiO_2_ particles reaggregated, returning to their initial state. This diffusion‐aggregation cycle was highly repeatable (Figure 3B). The kinetics of the diffusion‐aggregation process under various conditions are summarized in Figure 3C. The diffusion rate of Cu‐TiO_2_ was more than five times faster than the aggregation rate under all tested conditions, attributed to the enhanced electrolyte concentration gradient established under light illumination [32]. At different H_2_O_2_ concentrations, the diffusion rate of Cu‐TiO_2_ showed a positive correlation with the H_2_O_2_ concentration (Figure 3C_1_), consistent with faster formation of electrolyte and non‐electrolyte gradients at higher H_2_O_2_ concentrations, which drive more rapid movement. Notably, the aggregation speed did not continuously increase with increasing H_2_O_2_ concentration. At higher H_2_O_2_ concentration, the aggregation speed of Cu‐TiO_2_ decreased (Figure 3C_1_), which may be attributed to the stronger H^+^/O_2_ generation and outward concentration gradients induced by excessive H_2_O_2_, leading to greater particle dispersion and a longer reaggregation time after the light was switched off. The influence of light power density on the diffusion–aggregation behavior of Cu‐TiO_2_ was further investigated (Figure 3C_2_). The results showed that the diffusion speed of Cu‐TiO_2_ increased with increasing light power density, indicating its pronounced photoresponsive behavior. Notably, when the light power density increased from 933 to 1000 mW cm^−2^, the diffusion speed further increased whereas the contraction speed decreased, which may be related to the enhanced photocatalytic reaction and intensified local fluid disturbances under excessive irradiation. Moreover, under 1.0 wt.% H_2_O_2_, the diffusion speed of Cu‐TiO_2_ was highly dependent on the wavelength (Figure 3C_3_): under ultraviolet (UV) light (365 nm), the diffusion speed reached 2.72 µm s^−1^, whereas under green light (560 nm), it dropped to 0.32 µm s^−1^, suggesting that Cu‐TiO_2_ has almost no photoresponse in the green light region. Compared with commercial TiO_2_ or directly synthesized TiO_2_, MIL‐125(Ti)‐derived M‐TiO_2_ exhibits a pancake‐like morphology with a rough surface, which exposes more accessible active sites and facilitates the contact of H_2_O_2_ or pollutants with catalytic centers. To screen the optimal photocatalytic component, commercial P25, as‐prepared spherical TiO_2_‐s, and a series of MIL‐125(Ti)‐derived M‐TiO_2_ samples were compared. As shown in Figure 3D,E, the diffusion velocity of undoped TiO_2_ was 2.02 µm s^−1^. With increasing Cu doping, the diffusion velocity gradually increased and reached a maximum value of 2.72 µm s^−1^ at 1.5 at% Cu, denoted as Cu‐TiO_2_. This was mainly attributed to the trapping effect of Cu^2+^ on photogenerated electrons, which suppressed electron‐hole recombination and improved photocatalytic performance. However, when the Cu doping level exceeded 2.0 at%, excessive Cu may cause a shielding effect, reducing light penetration to the active sites and thus decreasing the diffusion velocity. In contrast, commercial P25, spherical TiO_2_‐s, and Fe‐, Ni‐, Mn‐, or Zn‐doped M‐TiO_2_ showed lower diffusion velocities, confirming the advantage of MOF‐derived Cu‐TiO_2_ in H_2_O_2_ decomposition and O_2_ generation. In addition, the diffusion or aggregation behavior of M‐TiO_2_ reflects its light‐responsive behavior and concentration‐gradient‐generating capability, and a higher diffusion velocity is beneficial for O_2_ generation, microvesicle‐like cavity formation, buoyancy regulation, and mass transfer.

### 2.3 Motion Study of SA/Cu‐TiO_2_/Fe_3_O_4_ Micromotors

**FIGURE 4:**
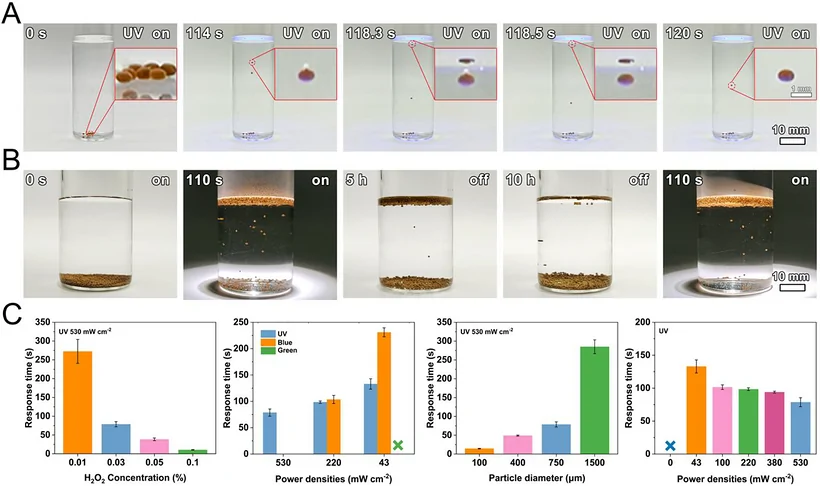
Light‐driven *Z*‐axis motion of SA/Cu‐TiO_2_/Fe_3_O_4_ micromotors (750 µm, 0.03 wt.% H_2_O_2_). (A) Surface bubble detachment on SA/Cu‐TiO_2_/Fe_3_O_4_ micromotors during light irradiation. (B) Formation of bubble‐induced microvesicles within SA/Cu‐TiO_2_/Fe_3_O_4_ micromotors under Xenon lamp illumination (510 mW cm^−2^), enabling buoyancy‐driven flotation. (C) Response time of SA/Cu‐TiO_2_/Fe_3_O_4_ micromotors under different conditions, defined as the time interval from initial light exposure to the onset of upward motion.

SA/Cu‐TiO_2_/Fe_3_O_4_ micromotors were prepared by the electrospraying technique (Figures S12 and S13). Remarkably, when Cu‐TiO_2_ was encapsulated within SA/Cu‐TiO_2_/Fe_3_O_4_ micromotors, the produced O_2_ accumulated inside the hydrogel matrix, leading to the formation of internal bubbles under illumination. Owing to the elasticity and semi‐permeability of the hydrogel, cyanobacterial‐like microvesicle structures were dynamically constructed along with the formation of O_2_ gas within micromotors. When the buoyancy force generated by the internal gas vesicles exceeded the gravitational force acting on the micromotors, the micromotors began upward *Z*‐axis motion (Figure 4A,B and Movies S4 and S5) [37]. We define the response time as the duration between the onset of illumination and the beginning of upward motion; a shorter response time indicates stronger photocatalytic performance. Within the uniformly distributed Cu‐TiO_2_ in the SA/Cu‐TiO_2_/Fe_3_O_4_ micromotor system, two types of O_2_ bubble formation were observed, both contributing to buoyancy. One type involved the adsorption of O_2_ bubbles on the surface of micromotors (Figure 4A). As shown in Movie S4 after 114 s of UV illumination, a large bubble formed on the micromotor surface, causing it to rise. By 118.3 s, the bubble reached the solution surface and detached at 118.5 s, after which the micromotors sank under the effect of gravity [38, 39]. Upon re‐illumination, surface O_2_ bubbles regenerated, enabling a periodic floating‐sinking cycle. However, this behavior was infrequently observed in our experiments and required continuous energy input, as similarly observed in previously reported systems [10, 11, 12]. More commonly, O_2_ bubbles generated inside the SA/Cu‐TiO_2_/Fe_3_O_4_ micromotors remained confined within the hydrogel matrix, forming stable cyanobacterial‐like microvesicles. As these vesicles were shielded from the external environment, they did not detach or collapse easily, allowing the micromotors to maintain stable flotation at the solution surface for extended periods. Over time in the dark, gradual gas exchange across the semi‐permeable SA matrix led to a slow decrease in buoyancy: after 10 h, a small amount of micromotors started to sink, and complete sinking occurred after more than 40 h. Notably, re‐exposure to light reproduced internal O_2_ within the microvesicles, restoring buoyancy and enabling the micromotors to refloat at the surface (Figure 4B and Movie S5).

As shown in Figure 4C, we quantified the response time of the SA/Cu‐TiO_2_/Fe_3_O_4_ micromotors under different conditions, including H_2_O_2_ concentration, light wavelength, micromotor size, and light intensity. Increasing the H_2_O_2_ concentration resulted in a shortened response time, attributed to the accelerated O_2_ production that rapidly enhanced buoyancy. Furthermore, micromotors exhibited markedly shorter response times under UV light (365 nm) compared to blue light (470 nm) when monochromatic light sources were employed. No upward motion was observed under green light (560 nm) irradiation, consistent with the poor photocatalytic performance of Cu‐TiO_2_ in the green light region, which was insufficient to produce O_2_ bubbles. Micromotor size also significantly influenced flotation behavior: as the diameter increased from 100 to 1500 µm, the response time varied from 5.3 to 120 s. This is attributed to the greater gravitational force acting on larger micromotors, necessitating more extensive O_2_ bubble generation to provide buoyancy. These results demonstrate that the cyanophyta‐inspired flotation behavior of SA/Cu‐TiO_2_/Fe_3_O_4_ micromotors can be finely modulated through environmental parameters and external light conditions.

### 2.4 Microvesicle Dynamics of SA/Cu‐TiO_2_/Fe_3_O_4_ Micromotors

The SA/Cu‐TiO_2_/Fe_3_O_4_ micromotors were designed to mimic the microvesicle structure of cyanobacteria. In this system, Cu‐TiO_2_ functions as the photocatalyst, generating gas within the hydrogel matrix to form the internal microvesicles, while *Z*‐axis motion is realized by regulating the gas‐liquid transport dynamics across the semi‐permeable membrane of the micromotors [40, 41]. Unlike natural cyanobacteria, the micromotor particle size was precisely controlled by adjusting the electrostatic spraying parameters, and the size of the internal microvesicles was modulated by tuning light intensity and illumination duration. To understand the mechanism of this biomimetic system, the dynamics of microvesicle formation and transmembrane mass transport were systematically investigated using high‐speed optical microscopy.

**FIGURE 5:**
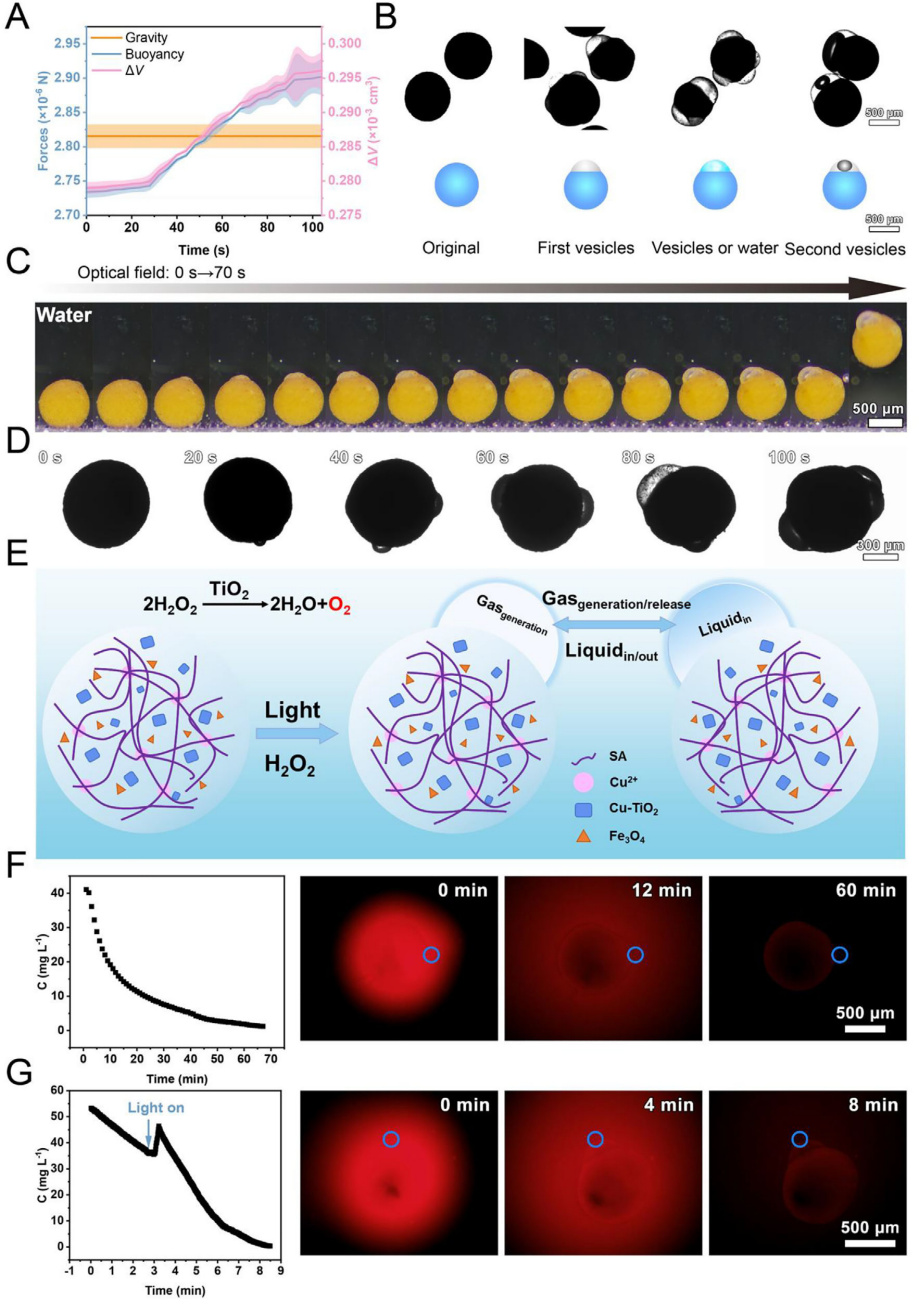
Microvesicle dynamic and transmembrane transport in SA/Cu‐TiO_2_/Fe_3_O_4_ micromotors. (A) Buoyancy evolution of SA/Cu‐TiO_2_/Fe_3_O_4_ micromotors under continuous illumination. (B) Schematic showing the four‐stage lifecycle of internal microvesicles: Stage I – pristine micromotor; Stage II – generation of the initial gas bubble, leading to the dynamic formation of microvesicles; Stage III – water infiltration and buoyancy reduction; Stage IV – regeneration of gas in the microvesicles under renewed illumination. Time‐dependent formation of microvesicles was captured via optical microscopy. (C) A high‐speed camera captures the formation and growth of microvesicles. (D) Preparation of micromotors containing microvesicles of different sizes. (E) The generation of microvesicles and the schematic representation of the transmembrane transport of substances. Fluorescence microscopy visualizing transmembrane transport in microvesicles: (F) passive diffusion under dark conditions and (G) Accelerated release under light‐induced bubble formation.

As shown in Figure 5A, during light exposure, the gravitational force acting on the SA/Cu‐TiO_2_/Fe_3_O_4_ micromotors was regarded as constant, while continuous generation of internal microvesicles progressively increased their buoyancy. After 50 s of UV illumination, buoyancy equilibrated with gravity; beyond this point, buoyancy exceeded gravitational force, triggering upward flotation (Movies S6 and S7) [37]. The quantitative relationship between the volume of a single micromotor and the corresponding buoyant force is provided in Equations S1 and S2. The buoyant force of the micromotors was primarily determined by the increased displaced volume induced by microvesicle formation. The increase in micromotor volume was accompanied by a corresponding increase in buoyant force. Indeed, the micromotor volume growth curve showed a similar trend to the buoyant force increase curve. As shown in Figure 5B, four distinct stages of microvesicle dynamics were identified in the SA/Cu‐TiO_2_/Fe_3_O_4_ micromotors: Stage I, micromotors in the original state, spherical and smooth in the absence of stimulation; Stage II, construction of the internal microvesicles under light exposure, leading to buoyancy‐driven *Z*‐axis motion; Stage III, gradual sinking during dark period due to the gas‐liquid exchange across the SA transmembrane, where increasing liquid infiltration into the microvesicles reduced buoyancy; and Stage IV, refloating after renewed light exposure, driven by regeneration of O_2_ within the microvesicles, restoring buoyant force (Movie S8) [42]. This floating‐sinking cycle can be repeated by controlling the light conditions. Dynamic microvesicle formation was clearly observed in SA/Cu‐TiO_2_/Fe_3_O_4_ micromotors exposed to 0.03 wt.% H_2_O_2_ solution under UV light (Figure 5C and Movie S7). As the illumination time increased, microvesicles appeared and progressively expanded. This process is governed not only by the Cu‐TiO_2_‐catalyzed generation of oxygen from H_2_O_2_ but also by the confinement and retention of the generated gas within the crosslinked sodium alginate hydrogel network, which promotes internal gas accumulation and the formation of stable microvesicles (Figure S14). Given the direct correlation between microvesicle size and micromotor buoyancy, precise control over microvesicle growth is critical for achieving tunable buoyancy regulation. As shown in Figure 5D, the microvesicle sizes at different irradiation durations were measured using an inverted microscope, demonstrating that microvesicle growth can be finely regulated by controlling the illumination conditions. (The average internal gas accumulation rates and external oxygen release rates under different illumination intensities are presented in Figure S15.)

To explore the mechanism underlying buoyancy regulation in SA/Cu‐TiO_2_/Fe_3_O_4_ micromotors, we proposed a schematic model (Figure 5E) illustrating the dynamic formation of internal microvesicles and the transmembrane transport of gas and liquid. To experimentally validate this concept, we conducted a fluorescence‐based assay to visualize the exchange of solutes through the semi‐permeable hydrogel matrix. Rhodamine B (RhB), a fluorescent dye, was used as a tracer to monitor liquid‐phase transmembrane diffusion (The relationship between fluorescence intensity and the concentration of the fluorescent dye RhB is shown in Figure S16). As shown in Figure 5F,G, micromotors were first incubated in RhB solution to fully load the microvesicles, followed by gentle rinsing to remove residual surface‐bound dye. The release of RhB from the micromotor interior to the surrounding medium was then tracked by fluorescence microscopy (Movie S9). Under dark conditions, RhB diffused outward gradually via passive diffusion, requiring approximately 60 min for complete release (Figure 5F). In contrast, upon light illumination, newly formed gas vesicles displaced the internal RhB solution more rapidly. This was reflected by a sudden increase in RhB fluorescence at 3.3 min, indicating initial release, followed by a sharp decline, with nearly complete expulsion within 8 min (Figure 5G). These results provide direct visual evidence of transmembrane mass exchange between the microvesicle interior and the surrounding solution, supporting the hypothesis that such transport processes modulate the buoyancy and dynamic flotation behavior of the micromotors.

### 2.5 3D Motion Manipulation of SA/Cu‐TiO_2_/Fe_3_O_4_ Micromotors

**FIGURE 6:**
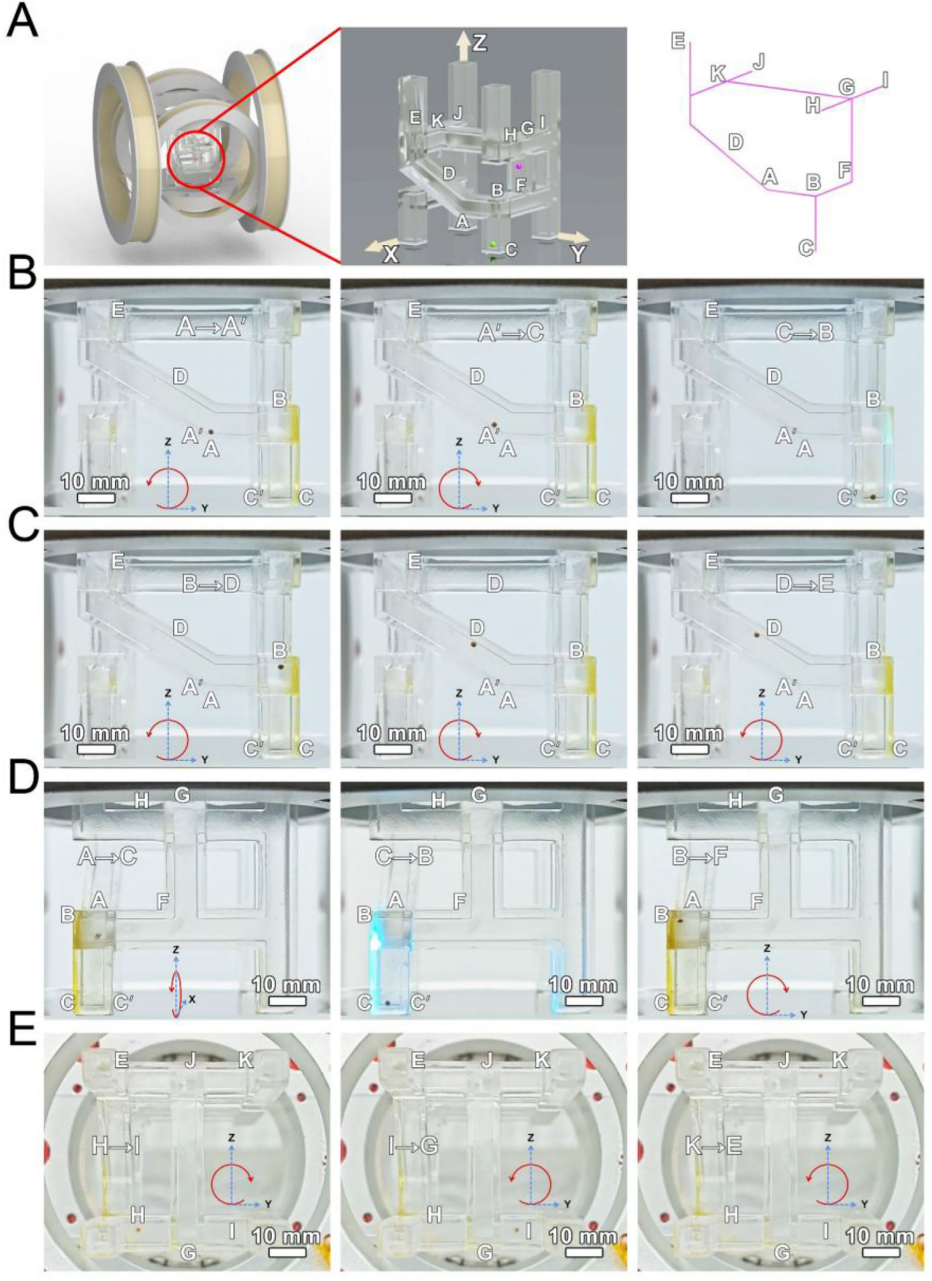
3D movement of SA/Cu‐TiO_2_/Fe_3_O_4_ micromotors under combined light and magnetic field stimulation. (A) Schematic of the experimental setup incorporating a magnetron coil for magnetic actuation. (B–E) Trajectories of micromotors in different planes: (B and C) X‐view, (D) Y‐view, and (E) Z‐view, demonstrating controllable 3D locomotion. Experimental conditions: [H_2_O_2_] = 0.03 wt.%, [magnetic field frequency] = 0.7 Hz.

In complex real‐world environments, MNMs often encounter obstacles. While natural cyanobacteria regulate their buoyancy to realize simple *Z*‐axis movement, they are unable to avoid obstacles during flotation. To enhance environmental adaptability, magnetic Fe_3_O_4_ NPs were incorporated into the SA/Cu‐TiO_2_/Fe_3_O_4_ micromotors, enabling magnetically controlled movement in the horizontal plane. The SA/Cu‐TiO_2_/Fe_3_O_4_ micromotors have a narrow hysteresis loop and a strong saturation magnetization of 7.04 emu g^−1^ (Figure S17A,B). Even under a weak external magnetic field, the micromotors achieved high magnetic induction [43], ensuring rapid and sensitive magnetic responsiveness. To demonstrate versatile motion control, SA/Cu‐TiO_2_/Fe_3_O_4_ micromotors were placed in custom‐designed S‐shaped tubes and 3D model structures (Figure 6, Figure S18, Movies S10 and S11). Their trajectories were directed by coordinated light irradiation and magnetic field manipulation.

As shown in Figure 6A, two different colored spheres indicate the position of the micromotors at different time points, representing two distinct motion paths: Route I (A→B→C→B→D→E) and Route II (A→B→C→B→F→G→H→I→G→K→J→E). In Route I, the micromotors were initially placed at point A. Magnetic field actuation alone was insufficient to propel the micromotor directly up the long‐inclined slope from A to D; only short‐distance movement (A→A') could be achieved. This limitation arose because, in rolling mode under magnetic actuation, the vertical component of the frictional force generated between the micromotors and the maze walls was unable to overcome gravitational force (Figure 6B). Upon reaching point C, the micromotor similarly could not advance to point B using magnetic field alone. However, following UV illumination, the formation of internal microvesicles generated buoyancy, enabling *Z*‐axis ascent to point B, where the micromotor achieved stable flotation. Subsequent magnetic field manipulation directed the micromotors horizontally to point D. At point D, in the absence of magnetic field input, the buoyancy and rolling friction force balanced, stabilizing the micromotor's position. If a reverse magnetic field was applied, the downward rolling friction force component was insufficient to overcome the buoyancy force, resulting in localized rolling without displacement [44]. Finally, under a controlled magnetic field, the micromotor successfully moved from point D to point E (Figure 6C). In Route II, the micromotor was first guided to point C by magnetic field actuation, then floated to point B under light‐induced buoyancy. It was subsequently maneuvered from point B to point F via magnetic field control, floated to point G by buoyancy (Figure 6D), and finally navigated from point G to point E through magnetic guidance (Figure 6E). These results demonstrate that the SA/Cu‐TiO_2_/Fe_3_O_4_ micromotors can be precisely controlled in complex 3D environments through the coordinated application of light and magnetic fields, thereby overcoming the limitation of natural cyanobacteria, which can only regulate movement along the *Z*‐axis.

### 2.6 Study on the Pollutant Removal by SA/Cu‐TiO_2_/Fe_3_O_4_ Micromotors

In recent years, MNMs have been widely explored for environmental remediation applications, particularly for the removal of chemical dyes and antibiotics, owing to their functional versatility and enhanced solute exchange capabilities. However, practical implementation remains challenging, as natural aquatic environments are often highly complex, containing abundant solid impurities such as silt, vegetation, and sand. Besides, in turbid water bodies, light intensity is significantly reduced due to the scattering effect, which impairs the motion and photocatalytic activity of the light‐controlled MNMs, thereby diminishing their pollutant removal efficiency or rendering them ineffective. Herein, inspired by the natural habitat of cyanobacteria, which perform daily vertical migrations to optimize access to light and nutrients in stratified lakes, we hypothesized that the self‐floating behavior of SA/Cu‐TiO_2_/Fe_3_O_4_ micromotors could overcome this limitation. Their buoyancy‐driven ascent would enable movement toward better‐illuminated regions even in turbid conditions, thus sustaining their photocatalytic activity. To evaluate this capability, we investigated the catalytic performance of SA/Cu‐TiO_2_/Fe_3_O_4_ micromotors against a range of representative pollutants, including industrial dyes (methyl orange, MO), fungicides (malachite green, MG), antibiotics (ciprofloxacin, CIP), and pesticide precursors (p‐nitrophenol, PNP).

For degradation performance assessment, a 300 W xenon lamp (350–1200 nm) was employed as the light source to investigate the photocatalytic activity of SA/Cu‐TiO_2_/Fe_3_O_4_ micromotors [45]. Silicon carbide (SiC, 40 nm) and sand (SiO_2_, 200 µm) were used as turbidants and insoluble impurities, respectively. A 70 mL contaminant solution was introduced into a vertical glass tube (length: 300 mm, diameter: 20 mm), with the xenon lamp positioned vertically above the tube at a fixed distance (detailed experimental procedures in the Supporting Information). SA/Cu‐TiO_2_/Fe_3_O_4_ micromotors were suspended in a highly turbid solution (SiC, 1.4 g L^−1^; 40 000 NTU) containing 0.03 wt.% H_2_O_2_. Solute transfer was facilitated by gentle stirring at 150 rpm, while vertical light irradiation was maintained from the tube opening. The motion of SA/Cu‐TiO_2_/Fe_3_O_4_ micromotors in a turbid water environment and the floating retention rate of the micromotor for 24 h were investigated in detail in Figures S19–S21A and Movies S12 and S13. The results showed that 750 µm SA/Cu‐TiO_2_/Fe_3_O_4_ micromotors exhibited good long‐term flotation stability after xenon lamp irradiation was removed, with a 24 h floating retention of 72.4%.

**FIGURE 7:**
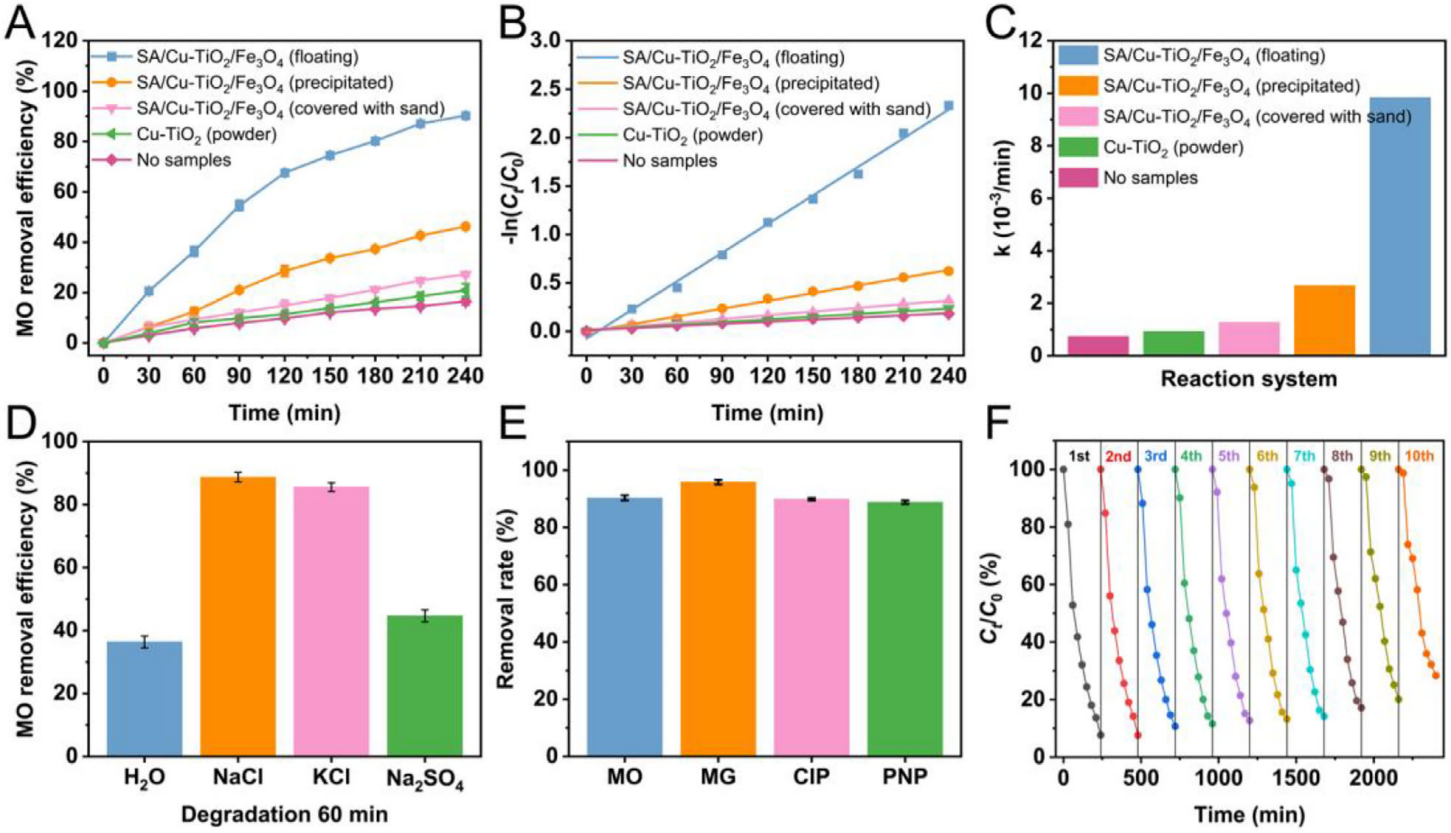
Pollutant removal performance of SA/Cu‐TiO_2_/Fe_3_O_4_ micromotors. (A) Comparison of methyl orange (MO) removal efficiency under different reaction conditions. (B) Pseudo‐first‐order kinetic plots of MO degradation [‐ln(*C_t_*/*C_0_*)]. (C) Apparent rate constants (*k*) for MO degradation under various micromotor states. (D) MO degradation performance of micromotors in different solution environments (e.g., NaCl, KCl, Na_2_SO_4_). (E) Removal efficiency of different pollutant types, including MG, CIP, and PNP. (F) Reusability of SA/Cu‐TiO_2_/Fe_3_O_4_ micromotors over ten cycles of MO degradation. Experimental conditions: [SA/Cu‐TiO_2_/Fe_3_O_4_ micromotors] = 0.3 g, [H_2_O_2_] = 0.03 wt.%, [SiC] = 0.14 wt.%, [MO] = 5 mg L^−1^, [NaCl] = [KCl] = [Na_2_SO_4_] = 1 mol L^−1^, [MG] = [CIP] = [PNP] = 10 mg L^−1^, [micromotor diameter] = 750 µm.

As shown in Figure S22, we compared the degradation effects of SA/Cu‐TiO_2_ and SA/Cu‐TiO_2_/Fe_3_O_4_ micromotors against MO. The SA/Cu‐TiO_2_/Fe_3_O_4_ micromotors exhibited superior degradation efficiency, which may be partially attributed to the additional Fenton‐like catalytic activity introduced by Fe_3_O_4_. Therefore, SA/Cu‐TiO_2_/Fe_3_O_4_ micromotors were used for further exploration of photocatalytic properties. Figure 7A summarizes the MO degradation performance under various conditions. The test environment consisted of MO (5 mg L^−1^), H_2_O_2_ (0.03 wt.%), and solid impurity (SiC NPs, 1.4 g L^−1^). When SA/Cu‐TiO_2_/Fe_3_O_4_ micromotors were either magnetically immobilized or fully buried by sand at the tube bottom, only 46.2% and 27.3% of MO were degraded within 240 min, respectively. This limited performance was mainly due to the weak light intensity at the bottom of the turbid solution, resulting in insufficient photocatalytic activation. In contrast, when the micromotors floated to the water surface, a 90.3% MO degradation rate was achieved within 240 min, an enhancement by a factor of 1.96 or 3.31 compared to micromotors resting at the bottom or buried under sand, respectively. The significantly improved performance is attributed to the stronger light exposure at the air–liquid interface, which promotes more efficient photocatalytic reactions. For comparison, when an equal amount of Cu‐TiO_2_ powder was used, the degradation effect after 240 min was only 20.9%, as the powder rapidly sedimented and experienced minimal light exposure. Consequently, the enhanced pollutant removal capability arises not only from the intrinsic photocatalytic activity of Cu‐TiO_2_ but also critically from the buoyancy‐enabled positioning of the SA/Cu‐TiO_2_/Fe_3_O_4_ micromotors at the illuminated surface. To quantitatively describe the degradation kinetics, the apparent reaction rate constant (*k*) was calculated using pseudo‐first‐order kinetic models. The floating SA/Cu‐TiO_2_/Fe_3_O_4_ micromotors exhibited a rate constant of 9.84 × 10^−3^ min^−1^, which was 3.68 and 10.58 times higher than that of sedimented SA/Cu‐TiO_2_/Fe_3_O_4_ micromotors (*k* = 2.67 × 10^−3^ min^−1^) and Cu‐TiO_2_ powder (*k* = 0.93 × 10^−3^ min^−1^), respectively. Collectively, these findings substantiate that the self‐floating behavior of SA/Cu‐TiO_2_/Fe_3_O_4_ micromotors is critical to achieving superior photocatalytic performance, offering a promising strategy for pollutant remediation in complex, turbid aquatic environments.

In practical applications, industrial textile wastewater typically contains complex mixtures of dyes and high concentrations of electrolytes [46]. To better simulate real‐world conditions, three different salts (NaCl, KCl, and Na_2_SO_4_, each at 1 mol L^−1^) were separately added to the MO solution. High‐ionic‐strength and high‐salinity environments may promote the buoyancy‐regulated motion of hydrogel micromotors (Figure S21B). Meanwhile, the micromotors maintained good pollutant removal performance in 1 mol L^−1^ NaCl, KCl, and Na_2_SO_4_ solutions (Figure 7D), indicating that the system retains stable pollutant removal capability under high‐salinity conditions. The presence of NaCl and KCl significantly enhanced the MO removal efficiency, reaching 88.71% and 85.57% within 60 min, respectively, more than four times higher than that observed in pure aqueous solution. In contrast, no noticeable change in MO removal efficiency was observed in the presence of Na_2_SO_4_. This difference is attributed to the role of Cl^−^ ions, which are known to facilitate the production of active species, thereby promoting the photocatalytic degradation process, as discussed in detail below. To further evaluate the versatility of SA/Cu‐TiO_2_/Fe_3_O_4_ micromotors for pollutant remediation, additional degradation experiments were conducted using other representative contaminants. As shown in Figure 7E, the floating SA/Cu‐TiO_2_/Fe_3_O_4_ micromotors achieved removal efficiencies of 95.83% and 89.87% for MG and CIP within 80 min under an aqueous environment (without added salt solution), respectively. In the case of PNP, the micromotors achieved a lower removal efficiency of 88.78% over 240 min. These results demonstrate the broad‐spectrum photocatalytic capability of the SA/Cu‐TiO_2_/Fe_3_O_4_ micromotors, highlighting their potential for efficient pollutant removal across a range of complex water environments. (The removal kinetics of MO, MG, CIP, and PNP by SA/Cu‐TiO_2_/Fe_3_O_4_ micromotors in different environments, as well as the absorbance standard curves of the contaminants, were investigated in detail in Figures S23–S25). As shown in Figure 7F, after ten consecutive cycles, the micromotors still maintained an MO removal efficiency of 71.62%, indicating their favorable cycling stability. Structural characterization showed that the micromotors retained an intact vesicular morphology after eight cycles of reuse; however, after ten cycles, partial rupture of the microsphere vesicular structure was observed (Figure S26A–C). In addition, the FTIR spectra of the micromotors before and after cycling showed no significant changes, suggesting that the MO degradation process did not cause noticeable chemical degradation of the micromotor matrix (Figure S26D). To further evaluate the performance of this system, we compared the SA/Cu‐TiO_2_/Fe_3_O_4_ hydrogel micromotors with representative bubble‐propelled micromotors reported for pollutant removal (Table S2). Although some reported systems exhibit faster apparent degradation kinetics, they usually require higher H_2_O_2_ concentrations or additional surfactants. In contrast, the present SA/Cu‐TiO_2_/Fe_3_O_4_ hydrogel micromotors operate at only 0.03 wt.% H_2_O_2_ and achieve efficient pollutant degradation under highly turbid‐water conditions. Therefore, the advantage of this system lies not in the fastest apparent degradation rate, but in its low chemical fuel input, buoyancy‐enabled migration toward the illuminated region, and magnetic recovery capability. In addition, we further compared this system with representative buoyancy‐driven micro/nanomotors in terms of bubble location, fuel or energy input, and quantitative buoyancy performance (Table S3). Compared with previously reported buoyancy‐driven systems that rely on continuous chemical fuel supply, pH/osmotic stimulation, enzymatic reactions, or photothermal gas expansion, the present SA/Cu‐TiO_2_/Fe_3_O_4_ hydrogel micromotors can retain photocatalytically generated O_2_ within the alginate hydrogel matrix and maintain 72.4% flotation after 24 h in the dark following light removal.

### 2.7 Removal Mechanism of SA/Cu‐TiO_2_/Fe_3_O_4_ Micromotors

Reactive oxygen species (ROS) produced during the photocatalytic reaction play a crucial role in the degradation of pollutants, including MO, MG, CIP, and PNP. To gain insight into the photodegradation mechanism, electron paramagnetic resonance (EPR) spectroscopy was employed. The ROS generation reactions involved are summarized in Equations (6)–(11) [47]:

(6) $$Ti^{4+}+H_{2}O_{2}\to Ti^{3+}+•{O_{2}}^{-}+2H^{+}$$

(7) $$Ti^{3+}+H_{2}O_{2}\to Ti^{4+}+•\mathrm{OH}+OH^{-}$$

(8) $$Cu^{2+}+H_{2}O_{2}\to Cu^{+}+•{O_{2}}^{-}+2H^{+}$$

(9) $$Cu^{+}+H_{2}O_{2}\to Cu^{2+}+•\mathrm{OH}+OH^{-}$$

(10) $$Fe^{3+}+H_{2}O_{2}\to Fe^{2+}+•{O_{2}}^{-}+2H^{+}$$

(11) $$Fe^{2+}+H_{2}O_{2}\to Fe^{3+}+•\mathrm{OH}+OH^{-}$$

**FIGURE 8:**
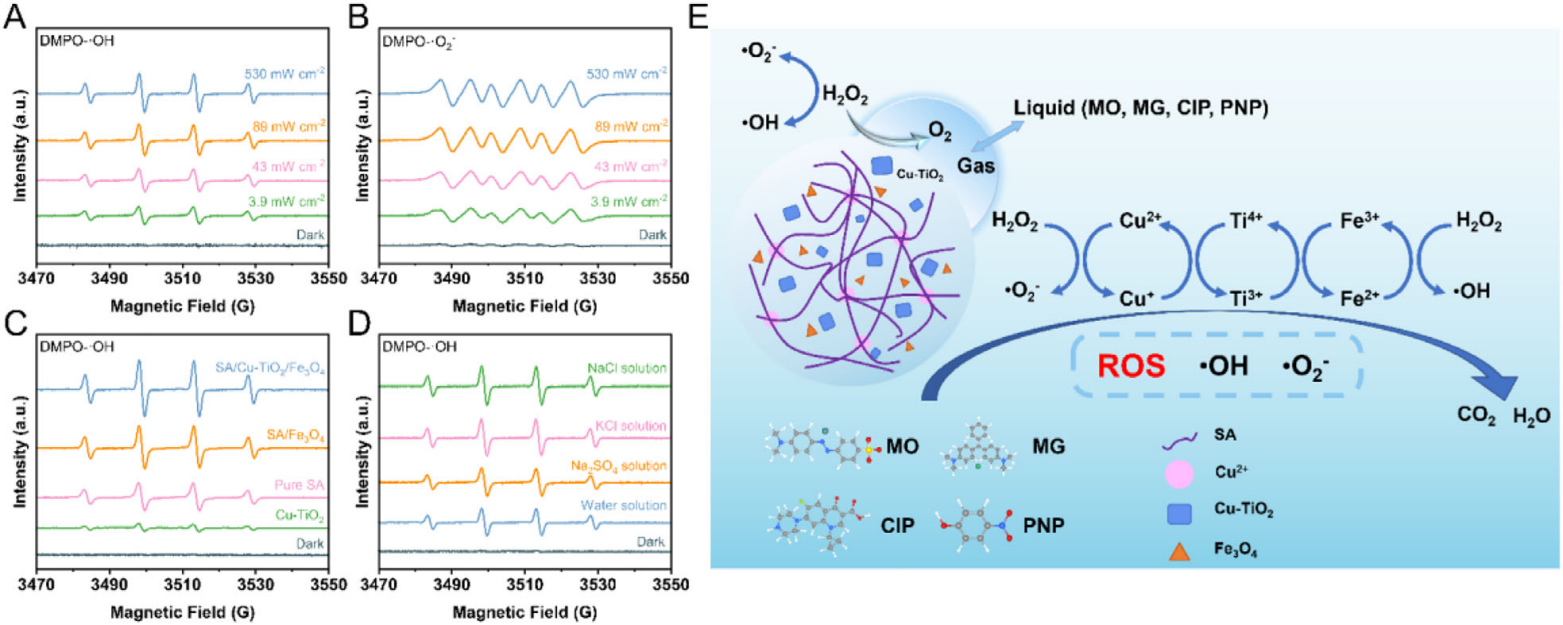
Mechanistic analysis of the MO degradation by SA/Cu‐TiO_2_/Fe_3_O_4_ micromotors. (A,B) EPR spectra of •OH and •O_2_^−^ radicals generated by SA/Cu‐TiO_2_/Fe_3_O_4_ micromotors under UV irradiation at different optical power densities. (C) Comparative EPR spectra of •OH radicals produced by different types of micromotors under UV light. (D) EPR spectra of •OH radicals generated by SA/Cu‐TiO_2_/Fe_3_O_4_ micromotors in different salt solutions (NaCl, KCl, Na_2_SO_4_). (E) Proposed schematic diagram of the photocatalytic degradation mechanism of MO by SA/Cu‐TiO_2_/Fe_3_O_4_ micromotors. Experimental conditions: [SA/Cu‐TiO_2_/Fe_3_O_4_ micromotors] = 10 mg, [Cu‐TiO_2_ catalyst] = 0.15 mg, [H_2_O_2_] = 0.03 wt.%, [NaCl] = [KCl] = [Na_2_SO_4_] = 1 mol L^−1^, [optical power densitie] = 530 mW cm^−2^, [micromotors diameter] = 750 µm.

The EPR spectra of ROS production under different conditions are shown in Figure 8A–D. In the absence of light, no detectable •OH or •O_2_^−^ signals were observed in the SA/Cu‐TiO_2_/Fe_3_O_4_ micromotor suspension, indicating that ROS generation is light‐dependent. Upon light exposure, both •OH and •O_2_^−^ signal intensities increased with higher light intensity (Figure 8A,B), with •OH showing a more significant enhancement, indicating that •OH is the dominant reactive species during photocatalysis [48]. The micromotors floating near the liquid surface, exposed to stronger light, produce more ROS, explaining their superior pollutant degradation efficiency compared to micromotors resting at the bottom. To identify the components responsible for ROS generation, we compared the •OH and •O_2_^−^ EPR signals of an equivalent amount of Cu‐TiO_2_ catalyst (0.15 mg) and SA/Cu‐TiO_2_/Fe_3_O_4_ micromotors (10 mg micromotors containing 1.5 wt.% Cu‐TiO_2_). Interestingly, the •OH signal intensity of the SA/Cu‐TiO_2_/Fe_3_O_4_ micromotors was 7.3 times higher than that of Cu‐TiO_2_ alone (Figure 8C), while their •O_2_^−^ intensities were nearly identical (Figure S27). This indicated that the additional sources beyond Cu‐TiO_2_ contributed to •OH generation. EPR analysis of SA micromotors cross‐linked with Cu^2+^ and SA/Fe_3_O_4_ micromotors revealed that both systems could independently generate •OH (Figure 8C). This is attributed to a Fenton‐like reaction between H_2_O_2_ and Cu^2+^ or Fe_3_O_4_ embedded within the hydrogel matrix [47]. Moreover, to explain the enhanced degradation observed in NaCl and KCl solutions, EPR spectra were compared under different salt conditions (Figure 8D and Figure S28). The •OH signal intensity was significantly stronger in NaCl‐ and KCl‐containing solutions. This enhancement is attributed to Cl^−^ ions facilitating more effective separation of photogenerated holes and electrons, thereby boosting the photocatalyst decomposition of H_2_O_2_ into active species [49]. The underlying photocatalytic mechanism of M‐TiO_2_ is further explained in Figures S29–S32.

Based on the above results, a comprehensive mechanism for the organic pollutant degradation by SA/Cu‐TiO_2_/Fe_3_O_4_ micromotors in aqueous environments is proposed (Figure 8E). Under light irradiation, Cu‐TiO_2_ embedded within the micromotors catalyzed the decomposition of H_2_O_2_, generating O_2_ and forming a microvesicle structure that drives buoyancy. Meanwhile, photoexcited Cu‐TiO_2_ produces active species, such as •OH and •O_2_^−^, in H_2_O_2_ solution. In parallel, the Cu^2+^ ions cross‐linked within the SA matrix and embedded Fe_3_O_4_ NPs facilitate additional •OH generation through a Fenton‐like reaction with H_2_O_2_. The combined action of light‐driven photocatalysis and Fenton‐like chemistry results in the continuous production of reactive oxygen species throughout the micromotor system, enabling highly efficient degradation of organic pollutants.

Finally, under light irradiation, the SA/Cu‐TiO_2_/Fe_3_O_4_ micromotors dynamically formed a microvesicle structure. These microvesicles not only provide buoyancy, enabling the micromotors to float toward the liquid surface where light intensity is stronger, but also facilitate pollutant uptake through transmembrane transport, thereby accelerating catalytic degradation. Given the extremely short lifetimes of ROS such as •OH and •O_2_^−^ (∼10^−9^ s) [50], proximity between ROS and pollutant molecules is essential for efficient degradation. The ability of pollutants to permeate into the interior of the microvesicles significantly shortens the diffusion distance between ROS and their targets, enhancing reaction kinetics. In summary, the synergistic interplay between microvesicles‐enabled flotation, electrostatic adsorption by the sodium alginate matrix, and localized ROS generation collectively promotes highly efficient pollutant removal in complex aqueous environments.

## 3 Conclusion

In this study, we developed SA‐based micromotors inspired by the microvesicle architecture of cyanobacteria. Metal‐doped photocatalysts M‐TiO_2_ (M═Cu, Fe, Ni, Mn, Zn) were first synthesized via solvothermal and high‐temperature calcination, among which Cu‐doped TiO_2_ (Cu‐TiO_2_) exhibited the fastest diffusion speed (1.62 µm s^−^^1^) under blue light in 1.0 wt.% H_2_O_2_. SA/Cu‐TiO_2_/Fe_3_O_4_ micromotors with tunable particle sizes were subsequently fabricated by electrostatic spraying, and their microvesicles' dimensions were precisely controlled by adjusting light intensity and exposure time. Fluorescence experiments revealed bidirectional transmembrane transport of substances within the micromotors, enabling dynamic buoyancy modulation and vertical (*Z*‐axis) motion. Even in low‐concentration H_2_O_2_ (0.03 wt.%), the micromotors ascended to the liquid surface and maintained a flotation retention rate of 72.4% after 24 h in the absence of light. In highly turbid water (40 000 NTU), the micromotors floated from the bottom to the surface within 120 s under weak light (3.7 mW cm^−2^), maintaining high photocatalytic efficiency. Under low‐speed stirring (150 rpm), the floating micromotors removed 90.3% of MO within 240 min, 1.96 times more efficient than sedimented micromotors. In saline conditions (1 mol L^−1^ NaCl), chloride ions enhanced charge separation, accelerating MO degradation to 91.5% within 60 min, a fourfold reduction in treatment time. This work demonstrates a controlled strategy to construct cyanobacteria‐inspired microvesicle systems and realize light‐activated, buoyancy‐regulated micromotor motion through gas/liquid transmembrane transport. The resulting micromotors exhibit exceptional pollutant degradation performance in turbid and complex aqueous environments, offering a promising platform for next‐generation environmental remediation technologies.

## 4 Experimental Section

### 4.1 Materials

Copper(II) chloride dihydrate (CuCl_2_·2H_2_O, AR), terephthalic acid (PTA, 99%), nickel chloride hexahydrate (NiCl_2_·6H_2_O, AR, 99%), manganese(II) chloride tetrahydrate (MnCl_2_·4H_2_O, AR, 99%), zinc sulfate heptahydrate (ZnSO_4_·7H_2_O, AR, 99.5%), copper(II) sulfate pentahydrate (CuSO_4_·5H_2_O, AR, 99%), methyl orange (C_14_H_14_N_3_NaO_3_S, 98%), rhodamine B (C_28_H_31_ClN_2_O_3_, AR), p‐nitrophenol (C_6_H_5_NO_3_, GC, >99%), ciprofloxacin (C_17_H_18_FN_3_O_3_, 98%), silicon carbide (SiC, 99.9%, 1–2 µm), titanium isopropoxide (TTIP, 97%) were purchased from Shanghai Macklin Biochemical Co., Ltd. Titanium butoxide (C_16_H_36_O_4_Ti, ≥99%), sodium alginate (SA, AR), malachite green chloride (C_23_H_25_ClN_2_, Biological Stain), iron chloride hexahydrate (FeCl_3_·6H_2_O, AR, 99%), iron (II, III) oxide (Fe_3_O_4_, 99.5%, 20 nm), dodecylamine (C_12_H_27_N, 98%) were purchased from Shanghai Aladdin Chemistry Co., Ltd. P25 (a commercial catalyst, AEROXIDE) was purchased from Evonik Operations GmbH. Ethanol (C_2_H_5_OH, AR) was purchased from Changshu Hongsheng Fine Chemical Co., Ltd. Acetonitrile (C_2_H_3_N, AR) was purchased from Wuxi Zhanwang Chemical Reagent Co., Ltd. N, N‐dimethylformamide (DMF, ≥99.5%) and methyl alcohol (CH_3_OH, ≥99.9%) were obtained from Hangzhou Shuanglin Chemical Reagent Co., Ltd. Sodium chloride (NaCl, AR), potassium chloride (KCl), and sodium sulfate (Na_2_SO_4_) were purchased from Hangzhou Gaojing Fine Chemical Industry Co., Ltd. Hydrogen peroxide (H_2_O_2_, 30%) was purchased from Shanghai Lingfeng Chemical Reagent Co., Ltd. All chemicals were procured from commercial suppliers and were used as received without further purification, except where specifically noted. Ultrapure deionized water (18.2 MΩ cm) was used throughout all the experiments.

### 4.2 Synthesis of M‐TiO_2_

MIL‐125(Ti_v_) was prepared according to a previous literature procedure [26]. Terephthalic acid (18 mmol) was dissolved in 54 mL of N, N‐dimethylformamide (DMF). Then, methanol (6 mL) and titanium butoxide (3.46 mmol) were added to the above mixture and stirred for 30 min to give a milky white mixture. Then transferred to a Teflon‐lined stainless‐steel autoclave at 130°C for 20 h. After cooling to room temperature, the suspension was centrifuged and then washed with DMF and methanol. Finally, MIL‐125(Ti_v_) was prepared by drying in an oven at 80°C for 12 h. 0.2 g of MIL‐125(Ti_v_) was homogeneously dispersed in 20 mL of deionized water. Subsequently, different mass ratios of metal salts were added, and the mixture was stirred at room temperature for 3 h. Afterward, the mixture was centrifuged, washed with deionized water, and dried in a vacuum oven at 80°C for 12 h to obtain M‐MIL‐125(Ti_v_). Subsequently, M‐MIL‐125(Ti_v_) was subjected to annealing in a muffle furnace at 450°C for a period of 4 h to obtain M‐TiO_2_ (M═Cu, Fe, Ni, Mn, and Zn).

### 4.3 Synthesis of TiO_2_‐s

The spherical TiO_2_‐s was synthesized based on previously reported methods [51]. Spherical titanium‐based precursors were synthesized via a sol–gel method. First, solution A was prepared by mixing 40 mL of absolute ethanol with 40 mL of acetonitrile at room temperature under magnetic stirring until a homogeneous solution was obtained. Subsequently, 0.36 g of dodecylamine and 0.4 mL of deionized water were added, and the mixture was continuously stirred until a clear and uniform solution was formed. Next, solution B was prepared by mixing 2 mL of acetonitrile with 6 mL of absolute ethanol, followed by the addition of 1.8 mL of TTIP. The mixture was gently shaken to ensure thorough mixing. To avoid rapid hydrolysis of TTIP, this step was carried out quickly under dry conditions.

Solution B was then rapidly poured into solution A under continuous stirring to initiate the reaction. To ensure homogeneity, care was taken to avoid the solution flowing slowly along the container wall. The resulting mixture was further stirred for approximately 2.5 h to allow sufficient hydrolysis and condensation of TTIP, forming a stable sol system. After the reaction, the products were collected by centrifugation (6000 rpm, 5 min) and washed several times with ethanol and deionized water to remove unreacted precursors and impurities. The obtained precipitate was then dried at 60°C for 12 h to yield the titanium‐based spherical precursor. Finally, the dried sample was calcined in air at 450°C for 2 h with a heating rate of 5°C min^−1^, resulting in structurally stable spherical TiO_2_, denoted as TiO_2_‐s, where “s” represents the spherical morphology.

### 4.4 Synthesis of SA/Cu‐TiO_2_/Fe_3_O_4_ Micromotors

The preparation of SA/Cu‐TiO_2_/Fe_3_O_4_ microspheres was done by the electrospraying method according to previous literature [27]. The Cu‐TiO_2_ (1.5 g) and Fe_3_O_4_ (0.5 g) were dispersed in 100 mL of deionized water, then sodium alginate (1.0 g) was added, and the solution was stirred in water at 60°C to obtain a homogeneous solution (electrospraying solution). Then, the electrospraying solution was introduced into the coagulant (0.1 m CuSO_4_) at a constant voltage and syringe pump advancement rate and kept for 3 h. Finally, SA/Cu‐TiO_2_/Fe_3_O_4_ microspheres were obtained by washing them with deionized water and then storing them in deionized water. The SA/Cu‐TiO_2_/Fe_3_O_4_ microspheres were immersed in a 0.03 wt.% H_2_O_2_ solution and subsequently exposed to a xenon lamp (300 W, 350–1200 nm) for a duration of 110 s. This resulted in the generation of microvesicles, which were then permitted to float and subsequently settle under their own weight. Following this process, the microspheres were designated as SA/Cu‐TiO_2_/Fe_3_O_4_ micromotors.

### 4.5 Characterization of SA/Cu‐TiO_2_/Fe_3_O_4_ Micromotors

The morphology of the samples was examined using a field emission scanning electron microscope (FESEM, Hitachi S‐4800, Japan). Energy‐dispersive X‐ray spectroscopy (EDS) analysis was performed using a Horiba EDX detector system integrated with the FESEM. Transmission morphology images and high‐resolution TEM images were collected employing a JEM‐2100 transmission electron microscope. The high‐angle annular dark‐field scanning transmission electron microscopy images for Cu‐TiO_2_ was obtained using JEM‐ARM300F equipment. The powder X‐ray diffraction (XRD) patterns were obtained using a Bruker Focus D8 diffractometer (Germany), equipped with a fine‐focus Cu sealed tube (λ = 1.54178 Å). The accelerating voltage and applied current were 40 kV and 80 mA, respectively. Fourier transform infrared (FTIR) measurements were performed on a Nicolet iS50 (USA) to investigate molecular vibrations and chemical bonds. X‐ray photoelectron spectroscopy (XPS) analysis was carried out on a Thermo Scientific ESCALAB Xi^+^ (USA) instrument fitted with a monochromatic Al Kα X‐ray source. UV‐Visible diffuse reflectance spectra (DRS) were recorded on an ultraviolet‐visible spectrophotometer (UH4150, Hitachi, Japan) with an integrating sphere attachment, using standard barium sulfate as a reference. Electron paramagnetic resonance (EPR) was performed on an A300‐10/12 spectrometer (Bruker BioSpin Co., Karlsruhe, Germany) to capture spin signals of reactive oxygen species. The motion behaviors of M‐TiO_2_ and the microvesicle formation process of SA/Cu‐TiO_2_/Fe_3_O_4_ micromotors were observed using an ICX41‐FLEDT inverted optical microscope (40x and 4x). The magnetic drive system of the SA/Cu‐TiO_2_/Fe_3_O_4_ micromotors consists of a 3D Helmholtz coil, which can be used to control magnetic forces in three orthogonal directions (x–y–z).

### 4.6 Analysis of Negative Phototactic Behavior of M‐TiO_2_

The motion behaviors of M‐TiO_2_ were observed using an ICX41‐FLEDT inverted optical microscope and a 40x objective lens, and a model BFS‐U3‐31S4M‐C camera was used for video acquisition at a frame rate of 30 fps. Finally, the motion velocity of M‐TiO_2_ was analyzed using the MtrackJ plug‐in in ImageJ software. To record the motion behaviors of M‐TiO_2_ in different environments, 1.0 mg of M‐TiO_2_ was first dispersed uniformly in 1 mL of H_2_O_2_ (0.1, 0.5, 1.0 wt.%, 2.0 wt.%), and then 100 µL of M‐TiO_2_ suspension was taken out and added dropwise onto a slide. The kinematic behaviors of M‐TiO_2_ were then observed using an inverted microscope under different wavelengths and different power densities of light conditions. The specific parameters of the light conditions were as follows: 365 nm UV light with a power density of 1.0 W cm^−2^; 470 nm blue light with a power density of 1.742 W cm^−2^; and 560 nm green light with a power density of 0.25 W cm^−2^.

### 4.7 Analysis of the Microvesicle Formation Process in SA/Cu‐TiO_2_/Fe_3_O_4_ Micromotors

To be able to study the formation of microvesicles at the microscopic level, an ICX41‐FLEDT inverted optical microscope and a 4x objective lens were used to observe the microvesicle formation process. The specific parameters were as follows: SA/Cu‐TiO_2_/Fe_3_O_4_ micromotors were placed on a slide, and then 100 µL of H_2_O_2_ (0.03 wt.%) solution was irradiated using UV light with a power density of 177 mW cm^−2^. Video acquisition was performed using an optical microscope at a frame rate of 10 fps to clearly observe the microvesicle formation process.

### 4.8 3D Motion of SA/Cu‐TiO_2_/Fe_3_O_4_ Micromotors

The micromotors can achieve precise motion in response to an external magnetic field. The micromotors are placed within a designed 3D labyrinth, and it is through the synergistic action of the external magnetic field and the microvesicles that complex motion is realized. Specifically, a 750 µm SA/Cu‐TiO_2_/Fe_3_O_4_ micromotor was placed within a 3D labyrinth containing H_2_O_2_ (0.03 wt.%), and the 3D maze was then placed in a 3D Helmholtz coil. The micromotors were made to float by applying light. The magnetic field strength in the 3D direction was then adjusted to control the planar movement of the micromotors. A smartphone was used for full video capture in both front and top views.

### 4.9 Transmembrane Transport Behavior of Substances in SA/Cu‐TiO_2_/Fe_3_O_4_ Micromotors

The transmembrane transport properties of microvesicles to dye solutions were investigated by means of a fluorescence test. Rhodamine B (RhB) was used as a fluorescent dye (green light can excite RhB to emit red light), and fluorescence biomicroscopy was used to observe the changes in RhB in microvesicles. The specific operational procedure was as follows: RhB transmembrane transport from the inside of the microvesicles to the outside; the micromotor was immersed in 10 mL of RhB (1000 mg L^−1^) for 1 h to ensure that the microvesicles were filled with RhB solution. Thereafter, a single micromotor was extracted, and the surface was meticulously cleansed of the RhB solution. The micromotor was then positioned within a slide containing 500 µL of H_2_O_2_ (0.03 wt.%) solution, which was then irradiated with green light (560 nm, 0.25 W cm^−2^). As a contrast, simultaneous application of UV light (365 nm, 530 mW cm^−2^) induced the expulsion of RhB by creating new bubbles in the microvesicles. The process was recorded using a model BFS‐U3‐31S4M‐C camera with a frame rate of 10 fps.

## Author Contributions

Y.Y., W. Z., and Q.W. contributed to the conception and writing of the paper. S.W. helped edit and discuss the paper. W.Z., Q.W., J.L., and K.L. performed all material syntheses, characterizations, data collection, and analysis. S.P. and J.P.‐L. discussed and organized the paper in depth, and participated in the writing of the paper. All authors read and commented on the paper.

## Conflicts of Interest

The authors declare no conflicts of interest.

## Supporting information




**Supporting File 1**: advs77408‐sup‐0001‐SuppMat.docx.

**Supporting File 2**: advs77408‐sup‐0002‐Movies.zip.

## Data Availability

The data that support the findings of this study are available from the corresponding author upon reasonable request.
