# Supplementary material for: MOF‐Derived Cu‐TiO2/Alginate Micromotors for Buoyancy‐Regulated 3D Motion
Source: Adv Sci (Weinh). 2026 Aug 27:e77408. Online ahead of print. doi: 10.1002/advs.77408 (PMC13521187; doi:10.1002/advs.77408)
Supplement: Supplementary file 1 [file ADVS-9999-e77408-s002.docx]

Supplementary Information

MOF-Derived Cu-TiO_2_/Alginate Micromotors for Buoyancy-Regulated 3D Motion

*Wei Zou,*^1, §^ *Qing Wu,*^1, §^ *Jiawei Lin,*^1^ *Kunchen Li,*^1^ *Yulong Ying,*^1^** Josep Puigmartí-Luis,^2,^ * Salvador Pané,^3,^ * Sheng Wang,*^1^***

^1^School of Materials Science and Engineering, Zhejiang Sci-Tech University, Hangzhou 310018, P. R. China, yingyulong@zstu.edu.cn

^2^ Departament de Ciència dels Materials i Química Física, Institut de Química Teòrica i Computacional, University of Barcelona (UB), Barcelona, 08028 Spain, josep.puigmarti@ub.edu

^3^Institució Catalana de Recerca i Estudis Avançats (ICREA), Pg. Lluís Companys 23, Barcelona 08010, Spain

^4^Multi-Scale Robotics Lab, Institute of Robotics and Intelligent Systems, ETH Zürich, Tannenstrasse 3, Zürich, CH-8092 Switzerland, vidalp@ethz.ch

^§^These authors contributed equally.

Supporting Information

Section S1: Materials and Methods

Section S2: Supplementary Figures S1-S32, and Table S1-S3

Section S3: Supplementary Movies S1-S13

Section S4: References (S1-S24)

1. Methods and Materials

1.1. Motion of SA/Cu-TiO_2_/Fe_3_O_4_ micromotors under light

To record the response rate of SA/Cu-TiO_2_/Fe_3_O_4_ micromotors in different conditions, 50 mg of micromotors were first placed in a glass tube with a diameter of 25 mm, and then 25 mL of H_2_O_2_ (0.01 wt%, 0.03 wt%, 0.05 wt%, 0.1 wt%) solutions with different concentrations were added. Subsequently, the motion process of the micromotors was recorded using a smartphone under light conditions of different wavelengths and different optical power densities. The specific light parameters were: 365 nm UV light with a power density of 530 mW cm^-2^; 470 nm blue light with a power density of 220 mW cm^-2^; and 560 nm green light with a power density of 43 mW cm^-2^.

In order to test the efficiency of different sizes of SA/Cu-TiO_2_/Fe_3_O_4_ micromotors floating on the liquid surface, 24 h of continuous video acquisition was performed using a smartphone. The specific parameters were: different sizes of SA/Cu-TiO_2_/Fe_3_O_4_ micromotors (300 mg) were placed in a 70 mL glass, and then 35 mL of H_2_O_2_ (0.03 wt%) solution was added. The efficiency of the micromotors floating on the surface of the solution was calculated by using a 300 W xenon lamp (350 nm-1200 nm, 510 mW cm^-2^) for 180 s of light and then left to stand for 24 h in the absence of light.

The same method was further applied to investigate the effect of high salt ion concentrations on the floating retention performance of SA/Cu-TiO_2_/Fe3O_4_ micromotors. Briefly, 750 μm SA/Cu-TiO_2_/Fe_3_O_4_ micromotors (300 mg) were used as the model system and separately placed in 70 mL glass vials. Subsequently, 35 mL of 0.03% H_2_O_2_ solution (control group) or 35 mL of 0.03% H_2_O_2_ solution containing 1 mol L^-1^ NaCl, KCl, or Na_2_SO_4_ (experimental groups) were added. After irradiation under identical conditions (300 W xenon lamp, 350-1200 nm, 510 mW cm^-2^) for 180 s to induce the floating of micromotors, the samples were maintained under dark conditions and continuously recorded for 24 h. The floating retention performance under high-salt conditions was evaluated by calculating the proportion of micromotors remaining at the liquid surface after 24 h.

**1.2.** **Microvesicle formation and photocatalytic O_2_ generation behavior of SA/Cu-TiO_2_/Fe_3_O_4_ micromotors under light irradiation**

To monitor the dynamic changes in microvesicle volume during their formation, the generation process was recorded in real time using a Shengchuang industrial magnifier. Briefly, 10 mg of SA/Cu-TiO_2_/Fe_3_O_4_ micromotors with an average diameter of approximately 750 μm were placed in a cuvette containing 3 mL of 0.03 wt% H_2_O_2_ solution and subsequently irradiated with ultraviolet light. Videos were recorded at a frame rate of 60 fps using the industrial magnifier, enabling clear visualization of microvesicle formation and the associated changes in volume.

The overall volume increase of the micromotors is primarily attributed to the formation of internal microvesicles. Therefore, the total volume of the micromotors can be approximated as the sum of the initial micromotor volume and the volume of newly formed microvesicles. Since the microvesicles generated by the micromotors exhibited a spherical-cap-like shape, their volume was calculated using the following equation:

$$\begin{aligned} \text{V=}\frac{\text{4}}{\text{3}}\text{π}\text{r}_{\text{1}}^{\text{3}}\text{+}\sum_{\text{i}\text{=1}}^{\text{n}} \frac{\text{πh}_{\text{i}}}{\text{6}}\left( \text{3}\text{r}_{\text{2, }\text{i}}^{\text{2}}\text{+}{\text{h}_{\text{i}}}^{\text{2}} \right) \#\left( \text{S}1 \right) \end{aligned}$$

where V is the total volume of the micromotor after vesicle formation, r_1_ is the radius of the original spherical micromotor, n is the number of generated vesicles, h_i_ is the height of the i-th spherical-cap-like vesicle, and r_2_, _i_ is the base radius of the i-th vesicle. According to Archimedes’ principle, the quantitative relationship between the microvesicle volume and buoyant force can be expressed as follows equation:

$$\begin{aligned} \text{F}_{\text{b}}\text{=}\text{ρgV}\#\left( \text{S}2 \right) \end{aligned}$$

where F_b_ is the buoyant force, ρ is the density of the surrounding liquid, g is the gravitational acceleration, and V is the displaced liquid volume corresponding to the micromotor after microvesicle formation.

Since the volume of newly formed microvesicles can be approximately regarded as the volume of accumulated internal oxygen, the variation in microvesicle volume was used to estimate the internal oxygen generation process. In addition, the fabricated micromotors exhibited similar sizes and masses, resulting in approximately identical gravitational forces required to achieve the floating state. Therefore, changes in illumination intensity mainly influenced the oxygen generation rate and the time required for micromotors to reach the floating state. Based on this assumption, the apparent oxygen generation rate of an individual micromotor was calculated using the following equation:

$$\begin{aligned} \text{r}_{\text{O}_{\text{2}}}\text{=}\frac{\text{V}_{\text{O}_{\text{2}}}}{\text{V}_{\text{m}}\text{t}}\#\left( \text{S3} \right) \end{aligned}$$

where $\text{r}_{\text{O}_{\text{2}}}$ is the apparent oxygen generation rate of an individual micromotor, $\text{V}_{\text{O}_{\text{2}}}$ is the oxygen volume estimated from the increase in microvesicle volume, $\text{V}_{\text{m}}$is the molar volume of gas, and t is the irradiation time required for the micromotor to reach the floating state.

To further evaluate the effect of light intensity on the O_2_ generation rate, the water-displacement method was used to quantify the O_2_ output released by SA/Cu-TiO_2_/Fe_3_O_4_ micromotors under different light intensities. Briefly, 300 mg of SA/Cu-TiO_2_/Fe_3_O_4_ micromotors with a diameter of approximately 750 μm were placed in a small sample vial, and 0.03 wt% H_2_O_2_ solution was added to fully immerse the micromotors. The sample vial was then placed inside a sealed reaction vessel containing 0.03 wt% H_2_O_2_ solution, with the external H_2_O_2_ solution level maintained above the opening of the sample vial to ensure liquid continuity between the inside and outside of the vial and eliminate residual air. A water-filled graduated cylinder was inverted directly above the sample vial in a water bath to collect the generated gas. UV light was used as the light source, and different light power densities were applied to investigate the O_2_ generation behavior of the micromotors. During irradiation, O_2_ generated from the catalytic decomposition of H_2_O_2_ by the micromotors directly entered the inverted graduated cylinder and was quantified from the displaced water volume. The gas volume was recorded at fixed time intervals, and the O_2_ generation rate was calculated from the change in O_2_ volume over time. The average O_2_ generation rate can be calculated using the following equation:

$$\begin{aligned} \text{r}_{\text{O}_{\text{2}}}\text{=}\frac{\text{V}_{\text{O}_{\text{2}}}}{\text{V}_{\text{m}}\text{Nt}}\boldsymbol{\#}\left( \text{S4} \right) \end{aligned}$$

Where $\text{r}_{\text{O}_{\text{2}}}$ is the average O_2_ generation rate per micromotor, $\text{V}_{\text{O}_{\text{2}}}$ is the collected O_2_ volume, $\text{V}_{\text{m}}$is the molar volume of gas, N is the number of micromotors, and t is the irradiation time.

1.3. Transmembrane transport of substances in SA/Cu-TiO_2_/Fe_3_O_4_ micromotors

Specific data processing for fluorescence determination of RhB concentration changes: Different concentrations of RhB solutions were prepared, and their fluorescence images were collected, and the fluorescence intensities (i.e., image brightness intensities) at different concentrations were calculated using Image J. The concentration-fluorescence intensity standard curve was obtained by fitting. The video of RhB transmembrane transport in microvesicles was processed in the same way to obtain the time-fluorescence intensity relationship, and then the standard curve was converted to obtain the time-RhB concentration relationship curve.

1.4. Photoelectrochemical measurements

To comprehensively assess the photoelectrochemical characteristics of M-TiO_2_, the CHI660E electrochemical workstation was employed as the testing instrument. These encompass electrochemical impedance test (EIS), photocurrent test (I-t), and model Schottky test (Mott-Schottky, MS). A 0.5 M Na_2_SO_4_ solution was employed as the electrolyte for the tests. A commercial indium tin oxide (ITO) conductive glass was utilized as the working electrode, a platinum sheet was designated as the counter electrode, and an Ag/AgCl standard electrode was selected as the reference electrode. The working electrode was prepared in accordance with the following procedure: 5 mg of M-TiO_2_ was homogeneously dispersed in a mixed solution consisting of 800 μL of deionized water and 200 μL of anhydrous ethanol. Thereafter, 60 μL of Nafion (5 wt%) solution was added to the mixture and ultrasonicated for 30 min. Thereafter, 300 μL of the mixture was added dropwise to the ITO glass electrode in five batches to form a 1 × 1 cm^2^ film, which was then dried naturally at room temperature. In the photoelectrochemical tests, a UV-LED laser (UVEC-4II) was used as the light source, and the specific light source parameters were as follows: 365 nm UV light with a power density of 530 mW cm^-2^, 470 nm blue light with a power density of 220 mW cm^-2^, and 560 nm green light with a power density of 43 mW cm^-2^.

1.5. Degradation performance of SA/Cu-TiO_2_/Fe_3_O_4_ micromotors on the pollutants

The simulated contaminants were initially degraded in a pure water environment. Methyl orange (5 mg L^-1^), malachite green chloride (10 mg L^-1^), ciprofloxacin (10 mg L^-1^), and p-nitrophenol (10 mg L^-1^) were utilized as the simulated pollutants; a glass tube with a diameter of 20 mm and a height of 300 mm was employed. This configuration was employed to load the simulated contaminants, thereby simulating the environment of an actual deep-water pool. The actual water environment is usually turbid, so 40 nm silicon carbide (SiC, 1.4 g L^-1^) was used as the turbidant and 200 μm quartz sand (SiO_2_, 7.1 g L^-1^) as the insoluble impurity. The turbidity of the whole system was measured to be 40000 NTU. Due to the high height of the glass tube, the overall contaminant transfer was poor, so low-speed stirring (150 rpm) was used to increase the contaminant transfer. The position of SA/Cu-TiO_2_/Fe_3_O_4_ micromotors in the solution was not affected by low-speed stirring. A xenon lamp was used as the photocatalytic light source, and in order to simulate the vertical irradiation of sunlight, the perpendicular distance between the xenon lamp and the liquid surface was kept at 150 mm, and the glass tube was wrapped with aluminum foil. Initially, 300 mg of SA/Cu-TiO_2_/Fe_3_O_4_ micromotors were dispersed in 70 mL of MO, MG, CIP, and PNP solutions containing SiC, respectively, and left in the dark for 3.5 h. This was done to reach adsorption-desorption equilibrium. The samples were then transferred to a glass tube, and 70 μL of 30 wt% H_2_O_2_ was added to keep the system at 0.03 wt% H_2_O_2_. Tests were carried out using a Shimadzu UV-1800 spectrophotometer in the wavelength range of 200-800 nm. The characteristic UV-Vis absorption peaks of MO, MG, CIP, and PN were 463 nm, 671 nm, 277 nm, and 317 nm, respectively, and the absorption values can be used to calculate the concentration of the pollutant solutions. The removal performance of pollutants under different conditions was investigated. Five groups were set up for control experiments with different conditions: (1) using a 300 W xenon lamp (350-1200 nm) to make the micromotors float on the surface of the solution for the photocatalytic reaction; (2) using a permanent magnet to immobilize the micromotors at the bottom of the glass tube, and then irradiating it with a 300 W xenon lamp (350-1200 nm) to simulate the treatment of water where the photocatalyst sinks to the bottom; (3) burying the micromotors in the bottom of the glass tube by adding 5 g of SiO_2_ at the bottom of the glass tube and then irradiated using a 300 W xenon lamp (350-1200 nm) to simulate water treatment under the condition that the photocatalyst was buried; (4) the photocatalytic reaction was carried out using an equal amount of Cu-TiO_2_ (4.5 mg) powder in a 300 W xenon lamp (350-1200 nm); (5) no SA/Cu-TiO_2_/Fe_3_O_4_ micromotors were added, only 70 mL (0.03 wt% H_2_O_2_) of MO solution containing SiC, and a 300 W xenon lamp (350-1200 nm) was used as a light source. Note that all degradation experiments were meticulously replicated three times. The resulting data were then subjected to rigorous analysis to ensure the reliability and accuracy of the findings.

Degradation of contaminants in a salt solution environment. 1 mol L^-1^ NaCl, KCl, and Na_2_SO_4_ were added to the contaminant solution, and the other steps were the same as in the pure water environment. The degradation efficiency (%) was determined based on Equation S5:

$$\begin{aligned} \text{Degradation efficiency }\left( \text{\%} \right)\text{=}\frac{\text{C}_{\text{0}^{\text{-}}}\text{C}_{\text{t}}}{\text{C}_{\text{0}}}\text{×100\%}\#\left( \text{S}5 \right) \end{aligned}$$

where *C*_0_ is the initial pollutant concentration used for the degradation test, and *C_t_* is the pollutant concentration in the solution at a given time. Rate constants (k) were calculated based on the pseudo-first-order kinetic model:

$$\begin{aligned} \text{-}\ln\frac{\text{c}_{\text{t}}}{\text{c}_{\text{0}}}\text{=}\text{k}_{\text{t}}\#\left( \text{S6} \right) \end{aligned}$$

2. Supplementary Figures

**Figure S1.** SEM and elemental content of M-MIL-125(Ti_v_).

**Figure S2.** SEM and elemental content of M-TiO_2_.

**Figure S3.** SEM and elemental content of hydrogel microspheres.

**Figure S4** TEM of P25 and TiO_2_-s

As shown in **Figure S5** and **Figure S6**, the lattice spacings of Cu-TiO_2_ were measured to be 0.35 nm and 0.18 nm, corresponding to the (101) and (200) crystal planes of TiO_2_, respectively, in good agreement with the selected area electron diffraction (SAED) patterns. MIL-125(Ti_v_), which contains Ti vacancies and is negatively charged, can electrostatically attract Cu^2+^ ions when immersed in a Cu^2+^ solution. Upon annealing, the Cu atoms become anchored within the TiO_2_ lattice, forming Cu-TiO_2_.^[^^1^^]^ A finer structure of Cu-TiO_2_ was investigated using high-angle annular dark-field scanning transmission electron microscopy (HAADF-STEM) (**Figure S6A-C**). Bright contrast spots were observed exclusively along the Ti atomic rows, attributed to the presence of Cu atoms occupying Ti vacancies.^[^^2^^]^ Line scan profiles across three randomly selected regions (blue lines, **Figure S6D-F**) further confirmed this observation: line 1 contained only Ti atoms, while lines 2 and 3 displayed both Ti and Cu signals. Additionally, STEM-EDS mapping (**Figure S6G**) verified the uniform distribution of the Ti, O, and Cu elements within the structure.^[^^1^^]^ provide direct evidence for the successful incorporation of Cu atoms into the TiO_2_ lattice and offers deeper insights into the compositional and structural integration of Cu-TiO_2_ following metallization.

**Figure S5.** A and B) TEM of Cu-TiO_2_. C) The selected area electron diffraction of Cu-TiO_2_.

**Figure S6.** Structure and micromorphology of Cu-TiO_2_. A) TEM of Cu-TiO_2_. B) HAADF STEM raw image of Cu-TiO_2_. C) Filtered HAADF STEM image from the area highlighted with a red rectangle in B and the corresponding line scan profiles. D-F) Line 1, Line 2, and Line 3 marked in (C). G) STEM-EDS mapping of O, Ti, and Cu of the fresh Cu-TiO_2_.

The structure of MIL-125 consists of a TPA ligand coordinated to cyclic Ti_8_ clusters, connected through triangular openings to form octahedral and tetrahedral cage geometries.^[^^3^^]^ In our synthesis, a reduced amount of titanium butoxide was used, leading to the formation of MIL-125 with Ti vacancies. The corresponding X-ray diffraction (XRD) patterns of the M-MIL-125(Ti_v_) and M-TiO_2_ are shown in **Figure S7**. The MIL-125(Ti_v_) presented characteristic diffraction peaks aligning well with previously reported literature,^[^^4^^]^ indicating the successful synthesis of MOF. After incorporation of metallized elements (M) at various concentrations and species into MIL-125(Ti_v_) (**Figure S7A-B**), the XRD patterns remained nearly identical to that of the pristine MIL-125(Ti_v_), indicating that the metal ions were uniformly dispersed within the framework without altering its crystallinity.^[^^5^^]^ Both P25 and TiO_2_-s exhibit characteristic diffraction peaks that are in good agreement with those reported in previous studies. Similar results were also obtained from the sintered samples, indicating that the introduction of metal species does not alter the crystalline phase of TiO_2_. The anatase crystal structure is well preserved in M-TiO_2_ (see **Figure S7C-D**).^[^^6^^]^

**Figure S7.** XRD of M-MIL-125(Ti_v_) and M-TiO_2_. A and B) M-MIL-125(Ti_v_). C and D) M-TiO_2_、P25 and TiO_2_-s.

Although HAADF-STEM and XRD analyses illustrate the presence of Cu atoms in Cu-TiO_2_ and demonstrate that Cu incorporation does not change the TiO_2_ crystal structure, these results alone are insufficient to definitively establish that Cu atoms occupy Ti vacancies. To further investigate the chemical state and coordination environment of Cu atoms, X-ray photoelectron spectroscopy (XPS) analysis was conducted. As shown in **Figure S8C**, the Cu 2p spectrum could be deconvoluted into four Gaussian peaks. The peaks at 952.4 eV and 932.6 eV are assigned to the Cu 2p_1/2_ and Cu 2p_3/2_ of Cu^2+^ species (90.51%, **Table S1**), respectively. The additional peaks at 951.0 eV and 931.4 eV are associated with Cu^+^ species (9.49%), suggesting that Cu atoms are chemically bonded to the TiO_2_ lattice rather than merely adsorbed on the surface. Furthermore, the O 1s spectrum (**Figure S8D**) displays a peak at 531.2 eV, attributed to oxygen species associated with Cu oxidation. This observation substantiates the formation of chemical bonds between Cu atoms and the TiO_2_ framework.^[^^7^^]^ **Table S1** shows the elemental content of Cu-TiO_2_.

**Figure S8.** A) Full XPS spectra of Cu-TiO_2_. B-D) High-resolution XPS spectra of (B) Ti 2p, (C) Cu 2p, (D) O 1s for Cu-TiO_2_.

Cu-TiO_2_ exhibits favorable photocatalytic characteristics. To evaluate its performance, the instantaneous photocurrent response (I-t) was assessed under different illumination conditions. All I-t curves showed significant and reproducible responses under UV (365 nm), blue (470 nm), and green (560 nm) light irradiation. Among these, Cu-TiO_2_ exhibited the highest photocurrent density under UV light, reaching 1.37 μA cm^-2^ at a UV intensity of 530 mW cm^-2^ (**Figure S9A-B**). This result indicates that Cu-TiO_2_ exhibited highly efficient photogenerated charge generation, separation, and transport. Increasing light intensity enhanced the photocurrent response across all wavelengths, reflecting an increase in photogenerated carrier density. Comparison of I-t curves for different samples (**Figure S9C-D**) demonstrated that Cu-TiO_2_ exhibited markedly higher photocurrent densities than TiO_2_ doped with other metal elements (Fe, Ni, Mn, Zn). Electrochemical impedance spectroscopy (EIS) analysis (**Figure S9E-F**) further confirmed that stronger illumination and shorter wavelengths (close to UV) resulted in smaller impedance arc radii, suggesting reduced electron transfer resistance and improved photocatalytic efficiency. Similarly, EIS spectra for various samples (**Figure S9, G and H**) showed that Cu-TiO_2_ had the smallest arc radius, consistent with its superior photocatalytic performance. Electrochemical tests conducted under identical light intensity (43 mW cm^-2^) (**Figure S10A-B**) also confirmed that the UV excitation produced a significantly higher photocurrent density compared to blue and green light. UV-Vis diffuse reflectance spectra (DRS) (**Figure S11A-B**) revealed that pure TiO_2_ exhibited minimal absorption beyond 400 nm, while M-TiO_2_ samples displayed enhanced absorption in the visible range. Cu-doped TiO_2_ exhibited notably stronger visible light absorption than pristine TiO_2_, characterized by a broad hump between 400 and 900 nm attributed to d–d transitions of Cu ions.^[^^1^^]^ This enhanced absorption explains the observable motion response of Cu-TiO_2_ under blue light and supports its superior diffusive mobility compared to other Cu doping levels.

**Figure S9.** Instantaneous photocurrent response (I-t) of M-TiO_2_. A) under different UV light intensities. B) under different light sources (UV, blue, green). C and D) instantaneous photocurrent response (I-t) of M-TiO_2_ under UV-light irradiation. E and F) Electrochemical impedance spectra (EIS) of Cu-TiO_2_ under varying illumination conditions. G and H) Electrochemical impedance spectra (EIS) of M-TiO_2_.

**Figure S10.** Electrochemical testing of Cu-TiO_2_ at UV light density of 43 mW cm^-2^. A) Instantaneous photocurrent response (I-t) of Cu-TiO_2_. B) Electrochemical impedance spectra (EIS) of Cu-TiO_2_.

**Figure S11.** UV-vis diffuse reflectance spectra (DRS) of M-TiO_2_. A) Cu_n_-TiO_2_. B) M-TiO_2_.

Electrospraying is the classic method for the preparation of microspheres. The particle size was controlled by tuning the key parameters of the electrostatic spraying process, including the SA concentration, feed rate, and applied voltage (**Figure S12A-C**). Increasing the SA concentration raised the solution viscosity, leading to larger microsphere diameters.^[^^8^^]^ The feed rate exhibited minimal influence on particle size once it exceeded 1.5 mL h^-1^. In contrast, voltage played a primary role in determining microsphere diameter: higher voltages led to smaller particle diameters.^[^^9-11^^]^ Optimal conditions for producing microspheres with a diameter less than 500 μm were established at an SA concentration of 1.0 wt%, a feed rate of 1.5 mL h^-1^, and an applied voltage of 12 kV. Incorporation of Cu-TiO_2_ slightly increased the particle diameter (**Figure S13A-B**), attributed to the increased SA viscosity from the particle addition. Nonetheless, microspheres with a diameter less than 100 μm could still be fabricated by fine-tuning the feed rate and voltage (feed rate 2 mL h^-1^, voltage 16 kV). SA/Cu-TiO_2_/Fe_3_O_4_ microspheres were subsequently fabricated by electrospraying a mixed solution of SA, Cu-TiO_2_, and Fe_3_O_4_ NPs. The addition of Fe_3_O_4_ NPs imparted magnetic responsiveness to SA/Cu-TiO_2_/Fe_3_O_4_ microspheres with diameters of 100 μm, 400 μm, and 750 μm (**Figure S13C**). These magnetically responsive microspheres are hereafter referred to as SA/Cu-TiO_2_/Fe_3_O_4_ micromotors.

**Figure S12.** The particle diameter of pure sodium alginate hydrogel microspheres. A) Sodium alginate concentration. B) Feed rate. C) Voltage.

**Figure S13.** The particle diameter of hydrogel microspheres. A) Cu-TiO_2_ concentration. B) Feed rate and voltage. C) Feed rate and voltage (contains 0.5 wt% Fe_3_O_4_).

To further clarify the effect of the alginate hydrogel network on gas retention, Ca^2+^- and Cu^2+^-crosslinked SA hydrogel sheets were prepared and characterized. As shown in **Figure S14A-B**, the Ca^2+^-crosslinked SA hydrogel exhibited a relatively irregular pore structure, with partial deformation and collapse of the pore walls. In contrast, the Cu^2+^-crosslinked SA hydrogel showed a more uniform, regular, and continuous honeycomb-like porous structure, indicating that Cu^2+^ crosslinking is more favorable for forming a hydrogel network suitable for gas confinement and mass transport.

Consistent with this observation, CaCl_2_-crosslinked SA/Cu-TiO_2_/Fe_3_O_4_ micromotors exhibited a lower floating ratio within 180 s than the Cu^2+^-crosslinked micromotors, as well as a higher settling ratio within 24 h, indicating reduced floating retention of the micromotors (**Figure S14C-D**). These results further confirm that the crosslinking ion and the resulting alginate network structure play important roles in O_2_ retention and buoyancy regulation.

**Figure S14.** Effect of crosslinking ions on the structure and buoyancy behavior of SA hydrogels and SA/Cu-TiO_2_/Fe_3_O_4_ micromotors. SEM images of SA hydrogels crosslinked with different metal ions. A) CaCl_2_-crosslinked SA hydrogel and B) CuSO_4_-crosslinked SA hydrogel. Floating and settling ratios of SA/Cu-TiO_2_/Fe_3_O_4_ micromotors crosslinked with C) Ca^2+^ and D) Cu²⁺. Experimental conditions for C and D:[micromotor diameter] = 750 μm.

To quantitatively investigate the effect of illumination intensity on gas generation behavior, the internal gas accumulation rate of SA/Cu-TiO_2_/Fe_3_O_4_ micromotors was estimated based on the time-dependent growth of microvesicles. Since the overall volume increase of micromotors mainly originates from the formation of internal gas-filled microvesicles, the variation in microvesicle volume can be used to approximate the accumulated gas volume. As shown in **Figure S15A**, the internal gas accumulation rate increased with increasing illumination intensity, leading to a shortened time required for the micromotors to reach the floating state. Furthermore, the oxygen release rate from the external environment was directly measured under different illumination intensities. As shown in **Figure S15B**, stronger illumination resulted in a higher oxygen release rate, confirming that enhanced light irradiation accelerated the Cu-TiO_2_-catalyzed decomposition of H_2_O_2_. These results demonstrate that illumination intensity regulates the gas generation process, thereby controlling the buoyancy-driven floating behavior of SA/Cu-TiO_2_/Fe_3_O_4_ micromotors.

**Figure S15.** Average O_2_ generation rates of SA/Cu-TiO_2_/Fe_3_O_4_ micromotors under different illumination intensities. A) Internal O_2_ generation rate estimated from microvesicle volume changes during the period from illumination onset to the critical floating state (tf). B) Oxygen release rate from the external environment was measured by the water-displacement method.

**Figure S16.** Fluorescence intensity versus concentration of the fluorescent dye RhB.

**Figure S17.** Magnetism of SA/Cu-TiO_2_/Fe_3_O_4_ micromotors. A) The magnetic hysteresis loops of SA/Cu-TiO_2_/Fe_3_O_4_ micromotors. B) Digital photograph illustrating the separation of SA/Cu-TiO_2_/Fe_3_O_4_ micromotors from the aqueous dispersion using a magnet.

The kinematic behavior of the micromotors was first evaluated using a customized S-shaped pipeline (**Figure S18** and **Movie S10**). Initially, the micromotors were placed at point A. A magnetic field was applied, guiding the micromotors along the pipeline to point B. Due to insufficient rolling friction between the micromotors and the inner wall of the tube to overcome the component of gravitational force, the micromotors rolled in place at point B without ascending. Upon light irradiation, the gas formation inside microvesicles generated sufficient buoyancy to lift the micromotors vertically from point B to point C. Subsequently, reapplication of a horizontal magnetic field propelled the micromotors from point C to the top of the S-tube at point D, demonstrating efficient 3D navigation under combined light and magnetic control.

**Figure S18.** Movement of SA/Cu-TiO_2_/Fe_3_O_4_ micromotors in an S-tube under light and magnetic stimulation.

Remarkably, the micromotors retained their buoyant behavior even in this turbid medium: after 120 s of light exposure, micromotors began floating upwards, and after 300 s, most micromotors had reached the liquid surface (**Figure S19A** and **Movie S12**). Optical power density measurements revealed that the light intensity at the bottom of the tube was only 3.7 mW cm^-2^, whereas at the liquid surface it reached 510 mW cm^-2^, an increase of approximately 137 times (**Figure S19B**). This result highlights that the turbid water environment drastically attenuates light transmission, yet the self-floating SA/Cu-TiO_2_/Fe_3_O_4_ micromotors can overcome this limitation. By migrating toward the more illuminated surface region, the micromotors enhance their access to light, significantly improving photocatalytic performance for environmental remediation under challenging conditions.

**Figure S19.** A) Screenshots of the floating motion of SA/Cu-TiO_2_/Fe_3_O_4_ micromotors illuminated by a xenon lamp in a turbid solution. B) Optical power densities at different positions.

To more visually demonstrate that low-speed stirring enhances mass transfer without affecting the kinematic behavior of the micromotors, a high-concentration MO solution (1500 mg L^-1^) was used to simulate contaminant diffusion under stirring conditions. As shown in **Figure S20** and **Movie S13**, low-speed stirring (150 rpm) did not disrupt the resting state of SA/Cu-TiO_2_/Fe_3_O_4_ micromotors initially sinking at the bottom. Without stirring, it takes 23 min for MO to diffuse from the bottom to the top of the glass tubes. In contrast, under low-speed stirring, the mass transfer time was reduced to 12 min, confirming that stirring significantly accelerates contaminant diffusion without perturbing micromotor flotation behavior.

**Figure S20.** A) Screenshot of the contaminant transfer process facilitated by low-speed stirring while SA/Cu-TiO_2_/Fe_3_O_4_ micromotors were floating. B) Time required for contaminant transfer from the bottom to the top.

The flotation kinetics of SA/Cu-TiO_2_/Fe_3_O_4_ micromotors with different sizes were further analyzed in **Figure S21A**. During the initial 60 s of light exposure, smaller micromotors (100 μm) exhibited faster flotation responses owing to their lower gravitational load. However, with prolonged illumination, larger micromotors (750 μm) generated larger volumes of internal microvesicles, resulting in enhanced buoyancy. After 180 s of continuous light exposure, 750 μm micromotors reached a 95% floating rate, whereas 100 μm micromotors reached only 50%, stabilizing in a sink-float equilibrium. Following light cessation and a 24 h standing period, the sedimentation rate of 100 μm micromotors reached 90%, whereas large micromotors had significantly higher flotation retention. Notably, 750 μm micromotors maintained a floating retention rate of 72.4%, attributed to the greater stability of their larger microvesicles. Based on these findings, 750 μm SA/Cu-TiO_2_/Fe_3_O_4_ micromotors were selected for subsequent pollutant degradation experiments. 750 μm SA/Cu-TiO_2_/Fe_3_O_4_ micromotors were selected to further investigate the effect of high salt ion concentrations on buoyancy dynamics, as shown in **Figure S21B**. High-salinity solutions enhanced the initial flotation of SA/Cu-TiO_2_/Fe_3_O_4_ micromotors by increasing the solution density. Moreover, the reduced swelling of the hydrogel network under high-ionic-strength conditions favored the stable retention of microvesicle-like cavities, thereby decreasing the sedimentation ratio and improving their long-term floating retention.

**Figure S21.** Floating retention of SA/Cu-TiO_2_/Fe_3_O_4_ micromotors after xenon lamp irradiation: A) different particle sizes; B) in different high-salt ion solutions. Experimental conditions for B: [salt] = 1 mol L^-1^, [micromotor diameter] = 750 μm.

**Figure S22.** Removal efficiency of MO by SA/Cu-TiO_2_ and SA/Cu-TiO_2_/Fe_3_O_4_ micromotors. The experiment conditions: [micromotors] = 0.3 g, [H_2_O_2_] = 0.03 wt%, [SiC] = 0.14 wt%, [methyl orange MO] = 5 mg L^-1^, [micromotors diameter] = 750 μm.

**Figure S23** shows the degradation kinetics of MG, CIP, and PNP using SA/Cu-TiO_2_/Fe_3_O_4_ micromotors. The micromotors exhibited superior degradation rates for MG and CIP, achieving over 90% removal within 80 min. Additionally, the degradation efficiencies of MG, CIP, and PNP were significantly enhanced when NaCl was introduced into the respective solutions, further confirming that both the SA/Cu-TiO_2_/Fe_3_O_4_ micromotors and Cl^-^ ions universally promoted pollutant degradation. To further distinguish between photocatalytic degradation and adsorption effects, experiments were conducted in pollutant solutions without H_2_O_2_ (**Figure S24**). Under these conditions, the micromotors demonstrated considerable adsorption capacities for MG and CIP, achieving removal efficiencies of 22.35% and 52.03% within 210 min, respectively. In contrast, minimal adsorption was observed for MO and PNP. This behavior is attributed to the anionic nature of sodium alginate, the primary matrix of the micromotors, which favors electrostatic adsorption of cationic species like MG, while exhibiting weak interactions with anionic dyes such as MO. Concentrations of MO, MG, CIP, and PNP during the degradation were calculated based on absorbance standard curves (**Figure S25**). Furthermore, the reusability of the SA/Cu-TiO_2_/Fe_3_O_4_ micromotors was evaluated through magnetic recovery.

**Figure S23.** Kinetic profiles of removal efficiency of SA/Cu-TiO_2_/Fe_3_O_4_ micromotors for different pollutants. (A) MO. (B) MG. (C) CIP. (D) PNP. The experiment conditions: [micromotors] = 0.3 g, [H_2_O_2_] = 0.03 wt%, [SiC] = 0.14 wt%, [methyl orange MO] = 5 mg L^-1^, [malachite green MG] = [ciprofloxacin CIP] = [p-nitrophenol PNP] = 10 mg L^-1^, [micromotors diameter] = 750 μm.

**Figure S24.** Adsorption removal efficiency of SA/Cu-TiO_2_/Fe_3_O_4_ micromotors for pollutants in a dark environment. The experiment conditions: [micromotors] = 0.3 g, [SiC] = 0.14 wt%, [methyl orange MO] = [malachite green MG] = [ciprofloxacin CIP] = [p-nitrophenol PNP] = 10 mg L^-1^, [micromotors diameter] = 750 μm.

**Figure S25.** Absorbance standard curves for different pollutants. A) MO. B) MG. C) CIP. D) PNP.

**Figure S26.** Characterization of SA/Cu-TiO_2_/Fe_3_O_4_ micromotors after ten consecutive cycles of MO degradation. A) Micrographs after three MO degradation cycles. B) Micrographs after eight MO degradation cycles. C) Micrographs after ten MO degradation cycles. D) FTIR spectra.

**Figure S27.** EPR spectra of •O_2_^-^ of SA/Cu-TiO_2_/Fe_3_O_4_ micromotors and Cu-TiO_2_.

**Figure S28.** EPR spectra of •OH of Cu-TiO_2_ micromotors in salt solution.

Cu-TiO_2_ serves as a principal photocatalyst in SA/Cu-TiO_2_/Fe_3_O_4_ micromotors. To further elucidate the photocatalytic mechanism, the precise band gap energy (Eg) values were calculated to reveal the production and transfer behavior of photogenerated carriers during pollutant removal. UV–Vis diffuse reflectance spectroscopy (DRS) measurements were used to construct a Tauc plot based on the Kubelka-Munk function (**Figure S29**). The findings indicate that pure TiO_2_ exhibits a band gap energy of 3.168 eV, consistent with literature values.^[^^12^^]^ Notably, Cu-doping led to a significant reduction in band gap energy, with Cu-TiO_2_ showing the narrowest band gap of 2.811 eV. Although the narrowed band gap enhances the light absorption capability of Cu-TiO_2_, its absorption in the green-light region remains insufficient to generate enough photogenerated carriers for efficient H_2_O_2_ decomposition. As a result, H^+^/O_2_ generation and concentration-gradient-driven propulsion are greatly weakened under green-light irradiation, leading to negligible micromotor motion. Further insights were gained from Mott–Schottky (MS) analysis (**Figure S30**-**S31**), where the MS curves exhibited a linear relationship with positive slopes, confirming that M-TiO_2_ is a typical n-type semiconductor. The flat band potential (E_FB_) of Cu-TiO_2_ was determined to be -0.9 V (relative to the Ag/AgCl reference electrode). According to the empirical formula Eg = E_VB_ (valence band) - E_CB_ (conduction band), the potentials of E_CB_ and E_VB_ (relative to the normal hydrogen electrode, NHE) are summarized in **Figure S32**.^[^^13^^]^ These results provide important insight into the energy band structure and charge separation capabilities of Cu-TiO_2_, which are critical for its enhanced photocatalytic performance.

**Figure S29.** Plots of the transformed Kubelka-Munk function versus the energy of light. A) Cu_n_-TiO_2_. B) M-TiO_2_.

**Figure S30.** Mott-Schottky curves of TiO_2_ with different Cu contents.

**Figure S31.** Mott-Schottky curves of M-TiO_2_.

**Figure S32.** Energy band structure and band gap diagram of M-TiO_2_.

3. Supplementary Tables

**Table S1.** The XPS peak area and atomic percentage of the different elements in Cu-TiO_2_.

| Catalyst | Elements | Peak area | Atomic (%) |
| --- | --- | --- | --- |
| Cu-TiO_2_ | Cu 2p | | |
|  | Cu^2+^ | 58218+30011 | 90.51 |
|  | Cu^+^ | 5382+3872 | 9.49 |
|  | Ti 2p | | |
|  | Ti^4+^ | 177864+87115 | 100 |
|  | O 1s | | |
|  | O-Ti | 205137 | 73.30 |
|  | O-Cu | 54367 | 19.44 |
|  | Vo | 20304 | 7.26 |

**Table S2.** Quantitative comparison of pollutant-removal efficiency and energy/fuel input between the present SA/Cu-TiO_2_/Fe_3_O_4_ hydrogel micromotors and representative bubble-propelled micromotors.

| **Material/system** | **Propulsion/ driving mode** | **Energy/fuel input** | **Removal performance** | **Main features/ limitations** | **Ref.** |
| --- | --- | --- | --- | --- | --- |
| SA/Cu-TiO_2_/Fe_3_O_4_ hydrogel micromotors | Photocatalytic O_2_ generation; buoyancy-regulated floating motion | 0.03 wt% H_2_O_2_; 163 mW cm^-2^ light irradiation | MO: 90.3% degradation within 240 min;  MG: 95.83% and CIP: 89.87% degradation within 80 min;  PNP: 88.78% degradation within 240 min | Low H_2_O_2_ concentration; buoyancy-enabled migration to illuminated region but requires light and H_2_O_2._ | This work |
| g-C_3_N_4_@carbon microsphere micromotor | Visible-light-induced bubble propulsion | 30% H_2_O_2;_ 250 mW cm^-2^ visible light | RhB: 95.88% degradation within 60 min | Visible light driven and high propulsion speed; relatively high H_2_O_2_ concentration | ^[^^14^^]^ |
| Ag/Pt-decorated glassy carbon bead micromotor | Self-propelled micromotor | 30% H_2_O_2_; no external light required | MB: 90.42% degradation within 30 min | High propulsion speed and autonomous floating/sinking; uses Ag/Pt noble metals and high H_2_O_2_ concentration | ^[^^15^^]^ |
| Pt single-atom/cluster-enhanced TiO_2_ Janus micromotor | Bubble propulsion generated by Pt-catalyzed H_2_O_2_ decomposition | 1 wt% H_2_O_2_; 1 wt% SDS; simulated solar irradiation | MO: >90% degradation within 160 min;  TCH: 90% degradation within 100 min; | Pt single atoms and clusters synergistically enhance photodegradation and H_2_O_2_ decomposition; still relies on Pt, H_2_O_2_, and SDS. | ^[^^16^^]^ |
| g-C_3_N_4_/Fe_3_O_4_ tubular micromotors | Photocatalytic propulsion (Visible light, H_2_O_2_) | 3 wt% H_2_O_2_ and 0.125wt% SDS; visible light | RhB: 99.8% degradation within 60 min | Exhibits linear and spinning motion; still depends on H_2_O_2_ and visible light. | ^[^^17^^]^ |
| rGO/ZnO/BiOI/Co-Pi/Pt micromotor | Visible-light photocatalysis and bubble propulsion | 3% H_2_O_2_; visible light | Rh6G: 94% degradation within 30 min | Fast degradation, but the structure is complex and contains Pt | ^[^^18^^]^ |
| Pt–ZnIn_2_S_4_ micromotors | Bubble propulsion generated by Pt-catalyzed H_2_O_2_ decomposition | 5 wt% H_2_O_2_; SDS; Visible light | MO: 97% degradation within 3 h; TCH: 70% degradation within 3 h | Pt enhances charge separation and LSPR effect; requires Pt, 5 wt% H_2_O_2_, SDS, and visible light | ^[^^19^^]^ |

**Table S3**. Quantitative comparison of representative buoyancy-driven micro/nanomotor systems.

| **Material/system** | **Gas/bubble location and buoyancy mechanism** | **Energy/fuel input** | **Quantitative buoyancy performance** | **Main features** | **Ref.** |
| --- | --- | --- | --- | --- | --- |
| SA/Cu-TiO_2_/Fe_3_O_4_ hydrogel micromotors | Internal retention of photocatalytically generated O_2_ in alginate hydrogel | 0.03 wt% H_2_O_2_; 163 mW cm^-2^ light irradiation | 72.4% flotation after 24 h in the dark | Long-term flotation after light removal; pollutant degradation; magnetic recovery | This work |
| CAT-PDPA@ZIF-L submarine-like MOF microparticles | Catalase-generated O_2_ bubbles regulated by pH-responsive PDPA | 0.25-2.0% v/v H_2_O_2_; pH stimulus | Vertical velocity: 1.59–5.17 mm s^-1^ | Reversible vertical motion; mainly for drug delivery. | ^[^^20^^]^ |
| Gas-encapsulating PLGA microcapsules (GEMs) | Internal gas bubbles generated by osmosis-induced cavitation | No H_2_O_2_; high concentration NaCl osmotic stimulus | >90% purity/recovery in buoyancy separation | Internal gas-bubble system; mainly for separation/purification | ^[^^21^^]^ |
| Urease-powered enzymatic nanomotor swarms | Urea decomposition lowers local density and induces solutal buoyancy | No H_2_O_2_; 100-300 mM urea | Vertical velocity: ca. 1.74-1.93 mm s^-1^ | Buoyancy-driven collective dynamics; mainly mechanistic study | ^[^^22^^]^ |
| Anti-CEA-CAT-ZIF-8 nanobots | Catalase-generated O_2_ bubbles retained on hydrophobic ZIF-8 | 0.1-7.5 mM H_2_O_2_, about 0.00034-0.0255 wt% | ~4 cm ascent within 4-30 min | Cell recognition/enrichment; not for pollutant degradation | ^[^^23^^]^ |
| PPy/ZIF-8 MOF micromotors | Stored gas in superhydrophobic ZIF-8 forms/expands bubbles | No H_2_O_2_; light-driven photothermal effect | Almost all reached the top within 20 min under blue light | Fuel-free self-floating; mainly for water sterilization | ^[^^24^^]^ |

4. Supplementary Movies

Movie S1: Diffusion-aggregation motion of Cu-TiO_2_ at different H_2_O_2_ concentrations under UV light with a power density of 933 mW cm^-2^.

Movie S2: Diffusion-aggregation motion of Cu-TiO_2_ under UV light at different power densities in 1.0 wt% H_2_O_2_.

Movie S3: Diffusion-aggregation motion of Cu-TiO_2_ under different light conditions in 1.0 wt% H_2_O_2_.

Movie S4: Z-axis motion of the micromotors under 0.03 wt% H_2_O_2_ and UV light with a power density of 530 mW cm^-2^.

Movie S5: Z-axis motion of the micromotors under 0.03 wt% H_2_O_2_ and 300 W xenon lamp with a power density of 510 mW cm^-2^.

Movie S6: The process of microvesicle generation in hydrogel microspheres under 0.1 wt% H_2_O_2_ and UV light with a power density of 177 mW cm^-2^.

Movie S7: The process of microvesicles generation in the micromotors under UV light.

Movie S8: The micromotors were used to generate new microvesicles in water-filled bubbles under 0.05 wt% H_2_O_2_ and UV light with a power density of 177 mW cm^-2^.

Movie S9: Fluorescence experiments on dye release from micromotors under natural and UV light conditions.

Movie S10: The movement of the micromotors in the S-tube is controlled by a combination of magnetic field and light.

Movie S11: The movement of the micromotors in a labyrinth tube is controlled by a combination of magnetic field and light.

Movie S12: The micromotors enable Z-axis motion under light conditions in turbid environments.

Movie S13: Low-speed stirring enhances mass transfer without affecting the kinematic behavior of the micromotors.

References

[1]	Y. Zhang, J. Zhao, H. Wang, et al., "Single-Atom Cu Anchored Catalysts for Photocatalytic Renewable H_2_ Production with a Quantum Efficiency of 56%," Nature Communications 13 (2022): 58.

[2]	L. Stidsholt, C. Scholz, U. Hermanns, et al., "Low Foraging Rates Drive Large Insectivorous Bats Away from Urban Areas," Global Change Biology 30 (2024): e17063.

[3]	Y. Han, W. Huang, M. He, et al., "Trace Benzene Capture by Decoration of Structural Defects in Metal-Organic Framework Materials," Nature Materials 23 (2024): 1531-1538.

[4]	Y. Sun, H. Ji, Y. Sun, et al., "Synergistic Effect of Oxygen Vacancy and High Porosity of Nano MIL-125(Ti) for Enhanced Photocatalytic Nitrogen Fixation," Angewandte Chemie 136 (2024): e202316973.

[5]	X. Song, Y. Wang, T. Zhu, et al., "Facile Synthesis a Novel Core-Shell Amino Functionalized MIL-125 (Ti) Micro-Photocatalyst for Enhanced Degradation of Tetracycline Hydrochloride under Visible Light," Chemical Engineering Journal 416 (2021): 129126.

[6]	Q. Cheng, Y.-J. Yuan, R. Tang, et al., "Rapid Hydroxyl Radical Generation on (001)-Facet-Exposed Ultrathin Anatase TiO_2_ Nanosheets for Enhanced Photocatalytic Lignocellulose-to-H_2_ Conversion," ACS Catalysis 12 (2022): 2118-2125.

[7]	M. Sun, W. Wan, X. Zhao, et al., "TiO_2_ Anchored Cu Single Atoms Catalysts Boost Nitrite Electroreduction to Ammonia for Water Remediation," Advanced Functional Materials 35 (2025): 2500553.

[8]	S. Rutkowski, T. Si, M. Gai, et al., "Hydrodynamic Electrospray Ionization Jetting of Calcium Alginate Particles: Effect of Spray-Mode, Spraying Distance and Concentration," RSC Advances 8 (2018): 24243-24249.

[9]	A. Partovinia, E. Vatankhah, "Experimental Investigation into Size and Sphericity of Alginate Micro-Beads Produced by Electrospraying Technique: Operational Condition Optimization," Carbohydrate Polymers 209 (2019): 389-399.

[10]	C. K. Ahenkorah, A. Zaitoon, L.-T. Lim, "Electrospraying of Submicron Alginate Beads in Canola Oil," Food Hydrocolloids 150 (2024): 109673.

[11]	C. Y. Karakaş, D. Özçimen, "A Novel Approach to Production of Chlorella Protothecoides Oil-Loaded Nanoparticles Via Electrospraying Method: Modeling of Critical Parameters for Particle Sizing," Biotechnol Appl Biochem 68 (2021): 659-668.

[12]	Y. Zhao, N. Yang, T. Zhou, et al., "Mechanism and Performance of Photocatalytic H_2_ Evolution for Carbon Self-Doped TiO_2_ Derived from MIL-125," International Journal of Hydrogen Energy 65 (2024): 151-157.

[13]	Y. Huang, Y. Zhu, S. Chen, et al., "Schottky Junctions with Bi Cocatalyst for Taming Aqueous Phase N_2_ Reduction toward Enhanced Solar Ammonia Production," Advanced Science 8 (2021): 2003626.

[14]	X. Song, Y. Tao, J. Liu, et al., "Photocatalytic-Induced Bubble-Propelled Isotropic G-C_3_N_4_-Coated Carbon Microsphere Micromotors for Dynamic Removal of Organic Pollutants," RSC Advances 12 (2022): 13116-13126.

[15]	J. Wu, H. Yu, W. Liu, et al., "Enhanced Degradation of Organic Pollutant by Bimetallic Catalysts Decorated Micromotor in Advanced Oxidation Processes," Journal of Environmental Chemical Engineering 10 (2022): 107034.

[16]	C. Lv, T. Wang, Y. Fang, et al., "Pt Single Atom and Atomic Cluster-Enhanced TiO_2_ Janus Micromotors for Efficient Bubble Propulsion and Photocatalytic Environmental Remediation," Inorganic Chemistry Frontiers 12 (2025): 2210-2223.

[17]	C. Zheng, X. Song, Q. Gan, et al., "High-Efficiency Removal of Organic Pollutants by Visible-Light-Driven Tubular Heterogeneous Micromotors through a Photocatalytic Fenton Process," Journal of Colloid and Interface Science 630 (2023): 121-133.

[18]	H. Zhou, B. Wu, L. Dekanovsky, et al., "Integration of BiOI Nanosheets into Bubble-Propelled Micromotors for Efficient Water Purification," FlatChem 30 (2021): 100294.

[19]	M. Yuan, M. Gong, H. Huang, et al., "Bubble-Propelled Plasmon-Reinforced Pt-ZnIn_2_S_4_ Micromotors for Stirring-Free Photocatalytic Water Purification," Inorganic Chemistry Frontiers 9 (2022): 5725-5734.

[20]	Z. Guo, T. Wang, A. Rawal, et al., "Biocatalytic Self-Propelled Submarine-Like Metal-Organic Framework Microparticles with pH-Triggered Buoyancy Control for Directional Vertical Motion," Materials Today 28 (2019): 10-16.

[21]	C. K. Yeh, Y. Huang, L. H. Schoenmakers, et al., "Tunable Gas Bubbles within Gas-Encapsulating Microcapsules (GEMs) for Buoyancy-Driven Purification," ACS Applied Materials &amp; Interfaces 17 31 (2025): 45130-45138.

[22]	S. Chen, X. Peetroons, A. C. Bakenecker, et al., "Collective Buoyancy-Driven Dynamics in Swarming Enzymatic Nanomotors," Nature Communications 15 (2024): 9315.

[23]	Z. Guo, C. Zhuang, Y. Song, et al., "Biocatalytic Buoyancy-Driven Nanobots for Autonomous Cell Recognition and Enrichment," Nano-Micro Letters 15 (2023): 236.

[24]	H. Huang, Y. Zhao, H. Yang, et al., "Light-Driven MOF-Based Micromotors with Self-Floating Characteristics for Water Sterilization," Nanoscale 15 (2023): 14165-14174.
